# A neurocutaneous Na_V_1.8 channelopathy underlies a genetic subtype of primary idiopathic hyperhidrosis

**DOI:** 10.1126/sciadv.aed3221

**Published:** 2026-07-17

**Authors:** Suguru Yamauchi, Jolien Vander Cruyssen, Michele Cervellera, Elizabeth Wohler, Jolien De Waele, Maxime Lammens, Corinne Boehm, Nara L Sobreira, Carley Blevins, Kaitlyn Ecoff, Yuping Mei, Margaux Theys, Ife Shoyombo, Maria Shishikura, Hongrui Yi, Kristen Rodgers, Beverly Lee, Hamza Khan, Andrei Gurau, Wasay Nizam, Shivani Shirodkar, Christine Kim, Joshua Choi, Daniel Coleman, Brooke Dorman, Takumi Iwasawa, Jin U Kang, Yves Heremans, Hajime Orita, Tetsu Fukunaga, Filip Van Petegem, Stephen B Baylin, Andreas S Barth, Tae Hwan Chung, Peter C Rowe, Glenn Treisman, Jinny Ha, Ruslan I Dmitriev, David Valle, Frank Bosmans, Malcolm V Brock

**Affiliations:** ^1^ Department of Surgery, The Sidney Kimmel Cancer Center, Johns Hopkins University School of Medicine, Baltimore, MD, USA.; ^2^ Department of Esophageal and Gastroenterological Surgery, Faculty of Medicine, Juntendo University, Tokyo, Japan.; ^3^ Molecular Physiology and Neurophysics Group, Department of Basic and Applied Medical Sciences, Faculty of Medicine and Health Sciences, Ghent University, Ghent, Belgium.; ^4^ Experimental Pharmacology, Department of Pharmaceutical Sciences, Faculty of Medicine and Pharmacy, Vrije Universiteit Brussel, Jette, Belgium.; ^5^ Tissue Engineering and Biomaterials Group, Department of Human Structure and Repair, Faculty of Medicine and Health Sciences, Ghent University, Ghent, Belgium.; ^6^ McKusick-Nathans Department of Genetic Medicine, Johns Hopkins University School of Medicine, Baltimore, MD, USA.; ^7^ Institute of Science, Tokyo, Japan.; ^8^ Whiting School of Engineering, Johns Hopkins University, Baltimore, MD, USA.; ^9^ Department of Vascular Surgery, Deborah Heart and Lung Center, Brown Mills, NJ, USA.; ^10^ Department of Surgery, Allegheny Health Network Cancer Institute, Pittsburgh, PA, USA.; ^11^ Department of Molecular Microbiology and Immunology, Johns Hopkins Bloomberg School of Public Health, Baltimore, MD, USA.; ^12^ Chicago College of Osteopathic Medicine, Downers Grove, IL, USA.; ^13^ Institute of Life Innovation Studies, Toyo University, Tokyo, Japan.; ^14^ Visual and Spatial Tissue Analysis (VSTA) core facility and Beta Cell Neogenesis Research Group (BENE), Genetics Reproduction and Development (GRAD), Faculty of Medicine and Pharmaceutical Sciences, Vrije Universiteit Brussel, Jette, Belgium.; ^15^ Department of Biochemistry and Molecular Biology, Life Sciences Centre, University of British Columbia, Vancouver, BC, Canada.; ^16^ Cancer Genetics and Epigenetics, The Sidney Kimmel Comprehensive Cancer Center at Johns Hopkins University, Baltimore, MD, USA.; ^17^ Department of Medicine, Division of Cardiology, Johns Hopkins University School of Medicine, Baltimore, MD, USA.; ^18^ Department of Physical Medicine and Rehabilitation, Johns Hopkins University School of Medicine, Baltimore, MD, USA.; ^19^ Division of Adolescent and Young Adult Medicine, Department of Pediatrics, Johns Hopkins University School of Medicine, Baltimore, MD, USA.; ^20^ Department of Psychiatry and Behavioral Sciences, Johns Hopkins University School of Medicine, Baltimore, MD, USA.; ^21^ Division of Thoracic Surgery, Department of Surgery, Johns Hopkins University School of Medicine, Baltimore, MD, USA.; ^22^ Center for Neurosciences (C4N), Faculty of Medicine and Pharmacy, Vrije Universiteit Brussel, Belgium.

## Abstract

Primary idiopathic hyperhidrosis (PIH) is a poorly understood disorder characterized by excessive sweating. We identify a genetically defined subset of PIH associated with rare coding changes in voltage-gated Na^+^ (Na_V_) channels. Whole-exome sequencing of hereditary PIH families revealed gene-level enrichment within the Na_V_ channel family, with *SCN10A* (Na_V_1.8) most strongly implicated. A knock-in mouse carrying the clinically observed Na_V_1.8^p.R14L^ substitution recapitulated excessive sweating. Na_V_1.8 was detected in a subset of postganglionic neurons in thoracic sympathetic ganglia in humans and mice, where p.R14L produced a gain-of-function profile that enhanced cholinergic responsiveness. Excessive sweating in mutant mice was reversible with Na_V_ channel inhibition, including clinically used agents and a Na_V_1.8-preferential compound. Together, these findings define a targetable neurocutaneous channelopathy underlying a subset of PIH and support a model in which excessive sweating arises from either gland-intrinsic dysfunction or altered sympathetic drive, motivating stratified therapeutic approaches.

## Teaser

A genetically defined Na_V_ channelopathy reclassifies primary idiopathic hyperhidrosis as a targetable neurocutaneous disorder.

## INTRODUCTION

Primary Idiopathic Hyperhidrosis (PIH) is a disabling disorder defined by established international consensus criteria (*1*) and characterized by excessive sympathetic cholinergic output leading to uncontrollable diurnal sweating. It affects an estimated 2–5% of the population (*2*–*4*), significantly impairs quality of life (*1*, *5*, *6*), yet remains poorly understood and historically classified as idiopathic (*7*), with no established molecular drivers or targeted therapies. PIH typically affects the palms, soles, and axillae and is often triggered by emotional stimuli. It is frequently accompanied by anxiety and other dysautonomic symptoms (*8*–*12*). Despite its prevalence and impact, PIH is often underreported due to social stigma and frequently mischaracterized as a cosmetic concern (*2*). Importantly, PIH is distinct from secondary hyperhidrosis, which can arise from physiological transitions, systemic illness or drug-induced effects (*13*).

Clinical and emerging mechanistic evidence support two non-mutually exclusive models of PIH. In some individuals, hyperhidrosis reflects a primary alteration in sweat gland morphology or function, where local sweat gland-targeted treatments such as botulinum toxin, topical glycopyrrolate, or iontophoresis, which are currently the only FDA-approved therapies for hyperhidrosis, provide substantial relief (*14*–*17*). However, a significant subset of patients fails to respond to local interventions and presents with normal sweat gland histology (*18*). In these cases, histological and neurophysiological studies implicate hyperactivity of the sympathetic nervous system (SyNS), particularly cholinergic postganglionic neurons (*19*). This form of PIH is clinically more challenging since FDA-approved therapies are incapable of durably reducing systemic sympathetic outflow. Moreover, it is plausible that both local and neuronal factors may contribute to disease expression in certain patients. Recognizing this spectrum, from gland-intrinsic to neuron-driven PIH, is crucial for guiding treatment: local therapies for gland dysfunction, and systemic interventions for sympathetic hyperexcitability. Within this framework, we focus on cholinergic postganglionic neurons that mediate sudomotor control. These neurons constitute only a small fraction (≤6%) of sympathetic neurons in rodent thoracic ganglia, and their abundance in humans remains uncertain (*20*–*24*). Their scarcity makes direct physiological interrogation challenging and motivates our combined genetic, imaging, and pharmacologic approach.

Emotional sweating is mediated by cortical–limbic activation that signals to preganglionic sympathetic neurons in the spinal cord, which synapse onto cholinergic postganglionic neurons releasing acetylcholine onto eccrine sweat glands (*19*, *25*, *26*). This circuitry is anatomically distinct from thermoregulatory sweating, which originates in hypothalamic preoptic nuclei in response to core temperature changes (*27*). The regional specificity and temperature independence of emotional sweating support the idea that PIH may arise from excessive excitability within cholinergic sympathetic pathways rather than primary gland pathology. Consistent with this interpretation, thoracic sympathectomy abolishes palmar hyperhidrosis and often alleviates associated dysautonomic symptoms (*28*, *29*), reinforcing a neurogenic contribution. Together, these clinical and physiological observations place PIH within a broader spectrum of disorders involving autonomic circuit dysfunction.

Approximately two-thirds of individuals with PIH report a positive family history, and the early age of onset and inheritance patterns suggest a Mendelian genetic basis (*30*, *31*). While candidate loci have been proposed (*19*, *30*, *32*–*35*), definitive molecular drivers remain elusive. Whole Exome Sequencing (WES) of multigenerational families revealed a significant enrichment of variants in voltage-gated Na^+^ (Na_V_) channel genes (*36*), with *SCN10A* (Na_V_1.8) emerging as the leading candidate. Na_V_1.8 has been extensively studied in the context of nociception (*37*, *38*) and implicated in cardiac syndromes associated with autonomic imbalance (*39*). It is expressed not only in sensory neurons (*40*) but also in intracardiac autonomic tissues (*41*), supporting a broader role in autonomic circuit regulation. Although hyperhidrosis has been reported in individuals carrying Na_V_1.8 mutations (*42*, *43*), its contribution to autonomic sweat disorders has not been mechanistically defined. In the families studied here, the Na_V_1.8^p.R14L^ substitution segregated with disease. Using a CRISPR/Cas9-engineered mouse model, we demonstrate that this variant enhances cholinergic responsiveness in postganglionic sympathetic neurons and produces excessive sweating that is reversible with Na_V_ channel inhibition. These findings define a genetically determined neurocutaneous channelopathy underlying a subset of PIH and provide a framework for mechanism-based stratification of patients.

## RESULTS

### Ion channelopathies as genetic drivers of PIH

We performed WES on probands from 32 multi-generational families with hereditary sweat disorders (see Methods, “Clinical evaluation of hyperhidrosis and autonomic dysregulation”). These individuals had not responded to local treatments, suggesting a systemic origin of disease. Across approximately 20,000 protein-coding genes analyzed, 398 (~2%) harbored rare functional variants (minor allele frequency ≤ 0.01) that appeared enriched in the PIH cohort relative to controls in initial screening. To prioritize genes for targeted statistical analysis, we chose 13 candidates (*SCN3A*, *SCN5A*, *SCN7A*, *SCN8A*, *SCN9A*, *SCN10A*, *SCN1B*, *SCNN1B*, *SCNN1D*, *LGALS3BP*, *ZFHX2*, *ZNF793*, *ZNF594*) based on: (*i*) biological plausibility, whereby genes encoding Na_V_ channels and auxiliary subunits were selected because of their known roles in neuronal excitability and nervous system function (*36*), and (*ii*) empirical observation, wherein genes showing recurrent rare variants within the PIH cohort (*e.g.*, *SCNN1B*, *SCNN1D*, *LGALS3BP*, *ZFHX2*, *ZNF793*, *ZNF594*) were included for enrichment testing. *ZFHX2* was selected based on prior linkage studies identifying 14q11.2–q13 as a susceptibility locus for primary palmar hyperhidrosis (*44*), with *ZFHX2* positioned within this interval. *ZNF793*, *ZNF594*, and *LGALS3BP* were incorporated due to known interactions with *ZFHX2* as documented in STRING (*45*). Of the 13 candidate genes examined, only *SCN10A* demonstrated statistically significant enrichment, showing rare variants (p.R14L, p.E825D, p.E852K, p.G1050E, p.R1268Q, p.C1288W) in 18.8% of hyperhidrosis cases (6/32) compared to just 6.8% in combined controls (82/1304), with consistently significant Fisher’s exact test/Chi-square analysis p-values across all comparisons (in-house controls p = 0.0155/0.0050, 1000 Genomes Project controls p = 0.0249/0.0117, and combined in-house and 1000 Genomes Project controls p = 0.0205/0.0082). These findings identified *SCN10A* as a potential contributor to PIH and, more broadly, suggested that altered sympathetic signaling may represent a convergent mechanism underlying disease susceptibility.

### Na_V_ channel variants contribute to PIH

**Fig. 1.**
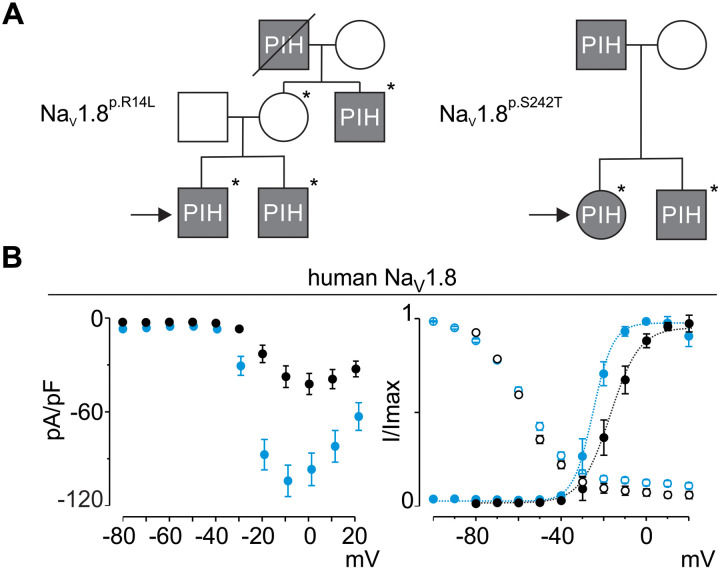
Na_V_1.8 variants identified in PIH families and functional impact of the p.R14L variant on channel gating. (**A**) Pedigrees of two multigenerational families affected by PIH showing segregation of *SCN10A* variants. In Family 1 (left), the proband (arrow) and affected relatives (gray-shaded symbols) carried the *SCN10A*^p.R14L^ variant (*). The maternal grandfather was not genotyped secondary to the lack of DNA. In Family 2 (right), the *SCN10A*^p.S242T^ variant was found in the proband (arrow) and a family member (*). The father was also affected but was not genotyped. (**B**) Whole-cell voltage-clamp analysis of human Na_V_1.8 wild-type (black) and p.R14L mutant channels (blue) expressed in ND7/23 cells. The p.R14L variant nearly doubled peak current density (control: *n* = 7, p.R14L: *n* = 8) and induced a hyperpolarizing shift in the voltage-dependence of activation (control: *n* = 5, p.R14L: *n* = 7), consistent with a gain-of-function phenotype.

*SCN10A* variants emerged as the most recurrent in our WES analysis, and affected individuals exhibited a striking phenotype. For example, one sequenced proband carried the *SCN10A*^p.R14L^ variant and presented with childhood-onset craniofacial and truncal hyperhidrosis, characterized by frequent, profuse sweating that necessitated multiple clothing changes a day and often restricted him to wearing only dark-colored garments (Fig. 1A). Pedigree and WES analysis revealed that *SCN10A*^p.R14L^ was also present in the proband’s maternal uncle with PIH, consistent with transmission through the maternal lineage. Although the maternal grandfather, who also exhibited excessive sweating, was not genotyped, he is the most likely source. The mother of the proband also carried *SCN10A*^p.R14L^ but did not report hyperhidrosis, suggesting incomplete penetrance. Structured assessment using the DN4 (*46*) and ASC-12 (*47*) questionnaires revealed features suggestive of neuropathic pain and allodynia in both the p.R14L carrier and his affected brother. These findings indicate sensory involvement without a dominant spontaneous pain presentation. Another sequenced proband in a 33rd family had childhood-onset palmar hyperhidrosis that intensified following an infection. Genetic analysis revealed the *SCN10A*^p.S242T^ variant, which is reported to be a gain-of-function mutation (*48*). Excessive sweating was also reported by her brother and father (Fig. 1A), suggesting familial inheritance. This familial pattern highlights a potential role of genetic modifiers in modulating phenotypic expression, as reported for other Na_V_ channel variants (*49*).

When transiently expressed in neuronal ND7/23 cells, electrophysiological analysis of human Na_V_1.8^p.R14L^ revealed a doubling of peak current density (from 52 ± 7 pA/pF to 106 ± 12 pA/pF; *P* < 0.01) and a hyperpolarizing shift in activation voltage (V_1/2_ from −16 ± 2 mV, *k* of 7 ± 1, to −25 ± 2 mV, *k* of 7 ± 1; *P* ≤ 0.01) (Fig. 1B and fig. S1). Na_V_1.8 protein sequences are 90% conserved between *Homo sapiens* and *Mus musculus* and the p.R14L locus is identical. Consistent with this conservation, mouse Na_V_1.8^p.R14L^ channels expressed in ND7/23 cells exhibited a near doubling of current density from a maximum of 50 ± 9 pA/pF to 94 ± 24 pA/pF (*P* < 0.01) and a hyperpolarizing shift in channel activation voltage (V_1/2_ from −15 ± 2 mV, *k* of 7 ± 1, to −22 ± 1 mV, *k* of 6 ± 1, *P* < 0.01) (figs. S1 and S2A). Because R14 is located within the N terminus, a region implicated in protein–protein interactions and broader regulation of channel function (*50*, *51*), this substitution likely perturbs early structural determinants of channel activity. Next, we used CRISPR/Cas9 genome editing to generate a Na_V_1.8^p.R14L^ knock-in mouse line which was bred to homozygosity and backcrossed for five generations to minimize possible effects of genetic background differences. Although mice lack the widespread eccrine gland distribution seen in humans, they possess eccrine sweat glands localized to their footpads. These glands are innervated by sympathetic neurons that switch postnatally from a noradrenergic to a cholinergic phenotype and respond primarily to cholinergic stimulation (*24*). Despite this anatomical restriction, the cholinergic-responsive footpads provided a physiologically relevant platform to assess how Na_V_1.8 drives sweating, establishing a model of pathogenic potential and positioning the Na_V_1.8^p.R14L^ variant as a framework for studying autonomic dysfunction in PIH.

### Na_V_1.8^p.R14L^ alters cholinergic responsiveness of sympathetic neurons

**Fig. 2.**
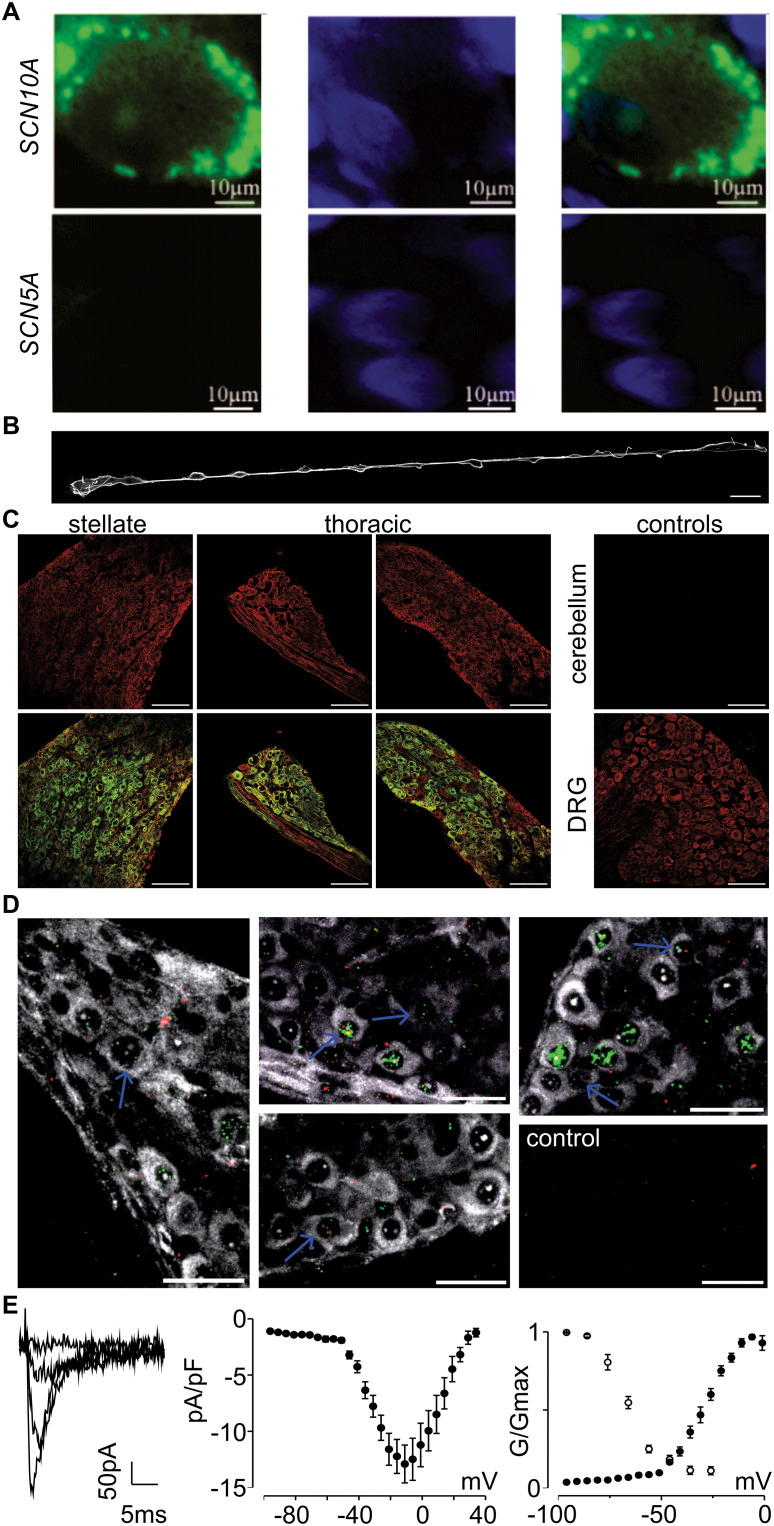
Na_v_1.8 is expressed in human and murine thoracic sympathetic ganglia. (**A**) Staining of human thoracic sympathetic ganglia shows Na_V_1.8 (green) and DAPI (blue), with no detectable Na_V_1.5. (**B**) Whole-mount epifluorescence imaging (DAPI) of the murine sympathetic chain (T1–T12) and stellate ganglion. Scale bar, 1 mm. (**C**) Immunohistochemistry on 15 μm cryosections of murine stellate and thoracic ganglia revealed Na_V_1.8 (red) colocalized (yellow) with TH (green). DRG and cerebellum were used as positive and negative controls, respectively. Scale bar, 100 μm. (**D**) RNA in situ hybridization in murine thoracic ganglia showed Na_V_1.8 (green) and ChAT (red) transcripts in Na_V_1.8-immunoreactive cells (grey) with blue arrows indicating the presence of both transcripts within a cell. Specificity was confirmed with control probes and secondary antibody-only staining. Scale bar, 30 μm. A wider field of the same preparation is shown in fig. S2D. (**E**) Whole-cell voltage-clamp recordings from murine thoracic sympathetic neurons revealed TTX-resistant Na^+^ currents. Left: representative trace. Middle: I–V plot (*n* = 9 from 6 biological replicates). Right: activation (*n* = 9 from 6 biological replicates) and inactivation (*n* = 6 from 4 biological replicates) curves. Incremental steps of 5 mV for 100 ms from −90 mV to 40 mV were used to assess activation. Inactivation was tested using a 500 ms pre-pulse with increasing voltage steps (10 mV/step) from −90 mV followed by a 100 ms pulse at −10 mV.

Na_V_1.8 expression is documented in DRG, intracardiac autonomic neurons, and the vagus nerve (*40*, *41*, *52*, *53*), and emerging evidence suggests its presence also in the SyNS (*41*, *54*–*57*). To substantiate these findings, we examined both human and mouse sympathetic tissues. Human tissue samples were obtained from surgical sympathectomies performed at Johns Hopkins Hospital as part of routine care for refractory ventricular arrhythmias. Immunostaining of paraffin-embedded human thoracic sympathetic ganglia with a knockout (KO)-validated Na_V_1.8 antibody (*58*) confirmed the presence of Na_V_1.8, whereas Na_V_1.5, the cardiac subtype, was not detected (Fig. 2A). In mice, we refined the anatomical dissection method described by Halder *et al.* (*59*) to isolate the full-length thoracic sympathetic chain (T1–T12), enabling intact whole-mount DAPI staining (Fig. 2B and fig. S3). Subsequent epifluorescence imaging revealed tyrosine hydroxylase (TH) (*60*) immunoreactivity throughout the chain, confirming the preservation and integrity of sympathetic ganglia (fig. S2B). Immunohistochemical analysis of permeabilized cryosections from mouse DRG (positive control), cerebellum (negative control), the stellate and thoracic ganglia, revealed Na_V_1.8 expression in both DRG and stellate/thoracic ganglia, with negligible signal in the cerebellum (Fig. 2, C and D, and fig. S2C), reinforcing the observation of Na_V_1.8 expression in the SyNS (*41*, *54*–*57*). Furthermore, orthogonal RNAscope analysis confirmed the presence of Na_V_1.8 transcripts in the stellate ganglion and thoracic sympathetic ganglia (T2–T12), co-localizing in a subset of cells with choline acetyltransferase (ChAT) mRNA, identifying the sudomotor-relevant cholinergic subset (Fig. 2D and fig. S2D). Finally, we measured Na_V_ channel-mediated currents in dissociated thoracic sympathetic neurons. Using 500 nM tetrodotoxin (TTX), we isolated putative Na_V_1.8-mediated currents (Fig. 2E), as both Na_V_1.8 and Na_V_1.9 are known to be TTX-resistant. The observed current kinetics were inconsistent with those of Na_V_1.9, which typically displays slower gating at 22°C (*61*). Instead, the biophysical properties of the TTX-resistant currents resembled those recorded from Na_V_1.8 channels expressed in DRG (*37*) with an activation and inactivation voltage V_1/2_ of −28 ± 1 mV (*n* = 6) and −64 ± 1 mV (*n* = 6), respectively, suggesting that Na_V_1.8 contributes to Na^+^ conductance in a subset of thoracic sympathetic neurons.

**Fig. 3.**
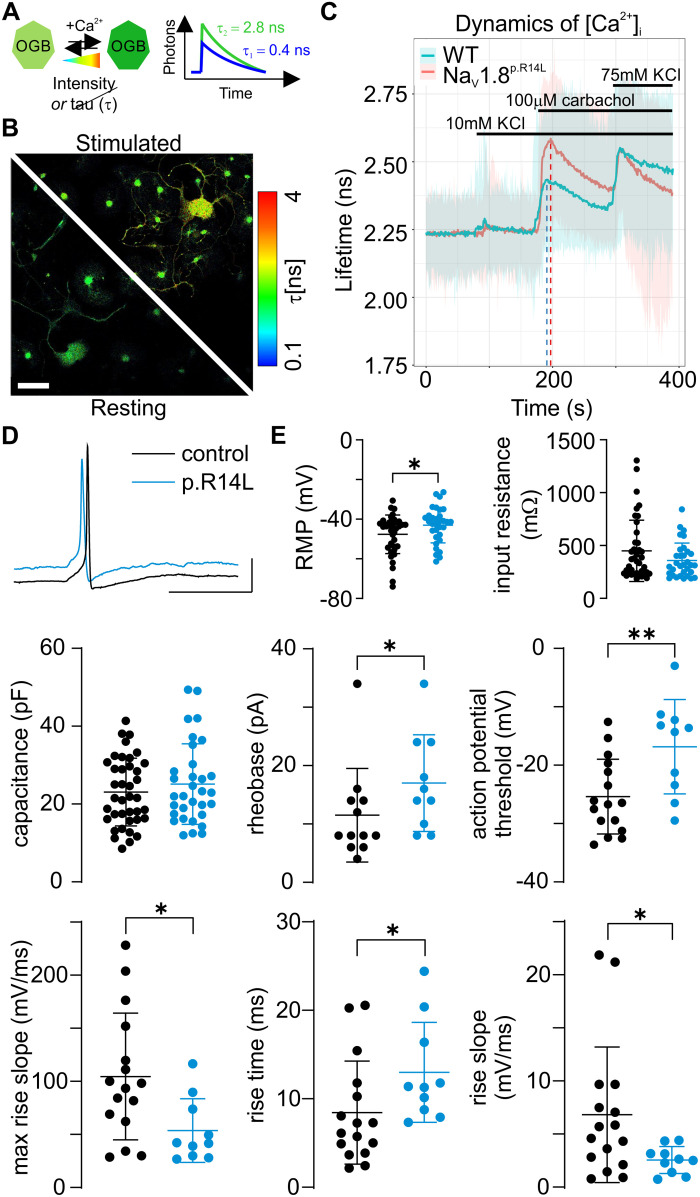
Dynamics of intracellular Ca^2+^ signaling and intrinsic membrane properties of WT and Na_V_1.8^p.R14L^ primary thoracic sympathetic neurons (T1–T12). (**A**) principle of FLIM-based Ca^2+^ sensing with Oregon Green BAPTA-1 a.m. (OGB-1) dye. (**B**) Examples of live Ca^2+^ FLIM images at resting and stimulated with 75 mM KCl conditions. Scale bar is 50 μm. (**C**) Combined single cell lifetime traces plot for WT and Na_V_1.8^p.R14L^ mutant mice primary thoracic sympathetic neurons. Difference between peaks (indicated with dashed lines): 0.15 ns, Wilcoxon test *P* value: 4.743e-180, Number of traces - Na_V_1.8^p.R14L^: 53, Number of traces - WT: 56. *n* = 3 biological replicates. (**D**) Representative action potential traces from WT (black) and Na_V_1.8^p.R14L^ (blue) neurons recorded in current-clamp mode. *X* axis is 100 ms, *y* axis is 10 mV. (**E**) Summary of intrinsic membrane properties. Input resistance and membrane capacitance were not significantly different between genotypes (WT: *n* = 39 cells; Na_V_1.8^p.R14L^: *n* = 32 cells). Na_V_1.8^p.R14L^ neurons displayed a depolarized resting membrane potential (WT: *n* = 39; Na_V_1.8^p.R14L^: *n* = 32), increased rheobase (WT: *n* = 12; Na_V_1.8^p.R14L^: *n* = 10), and a more depolarized action potential threshold (WT: *n* = 16; Na_V_1.8^p.R14L^: *n* = 10). In addition, mutant neurons exhibited reduced maximum rise slope, prolonged rise time, and decreased rise slope, consistent with altered spike initiation kinetics. Data are presented as mean ± SD (standard deviation). Statistical comparisons were performed using unpaired two-tailed Student’s *t*-tests or Mann–Whitney tests as appropriate. *P < 0.05; **P < 0.01.

Dissociated thoracic sympathetic cultures contain mixed postganglionic neuron types, and morphology does not reliably distinguish sudomotor from adrenergic neurons. Because cholinergic sudomotor neurons represent only a small fraction of the population, targeted manual patch-clamp recordings are intrinsically low-yield. We therefore started with Fluorescence Lifetime Imaging Microscopy (FLIM) as a population-level readout of genotype-dependent cholinergic responsiveness rather than a sudomotor-specific measure. FLIM enables functional assessment across multiple cells in parallel and provides precise measurements of intracellular Ca^2+^ dynamics while minimizing artifacts from dye loading, photobleaching, and intensity fluctuations introduced by manual stimulus application (Fig. 3, A and B, and fig. S4, A to D). Dissociated and cultured thoracic sympathetic neurons from wild-type (WT) and Na_V_1.8^p.R14L^ mice were loaded with the Oregon Green BAPTA-1 a.m. (OGB, 2.5 μM) dye for Ca^2+^ imaging. To probe cholinergic signaling across mixed sympathetic cultures in which cholinergic sudomotor neurons are rare, we leveraged the ability of FLIM to sample regions of interest (ROIs) in parallel and provide stable, quantitative data when applying sequential stimuli under standardized conditions: (i) 10 mM KCl as a depolarizing challenge to assess baseline excitability, (ii) 100 μM carbachol in Ringer solution to probe cholinergic signaling, and (iii) 75 mM KCl as a positive control for maximal cellular activation (movies S1, A and B, and S2, A and B). Sympathetic neurons from Na_V_1.8^p.R14L^ mice displayed significantly heightened Ca^2+^ responses to carbachol compared to WT, indicating amplified cholinergic signaling in postganglionic neurons (Fig. 3C). This hyperresponsiveness was evident in both peak fluorescence lifetime shifts and slower signal decay, while baseline and recovery values after the 10 mM KCl challenge showed a non-significant trend. Pooled analysis across three biological replicates confirmed the robustness of this exaggerated cholinergic response despite the low abundance of cholinergic neurons. These findings provided evidence that Na_V_1.8^p.R14L^ enhances carbachol-evoked responses in a subset of postganglionic sympathetic neurons.

To determine whether this functional hyperresponsiveness was reflected in intrinsic membrane properties, we performed current-clamp recordings from postganglionic sympathetic neurons, which did not reveal classical intrinsic hyperexcitability in p.R14L cells. Instead, Na_V_1.8^p.R14L^ neurons exhibited a modest depolarization of resting membrane potential (−47.6 ± 9.7 mV (*n* = 39) to −43.0 ± 9.0 mV (*n* = 32; p = 0.042), increased rheobase (11.5 ± 8.0 pA (*n* = 12) to 17.0 ± 8.3 pA (*n* = 10; p = 0.045), depolarized action potential threshold (−25.4 ± 6.4 mV (*n* = 16) to −16.8 ± 8.1 mV (*n* = 10; p = 0.006), and reduced action potential upstroke kinetics (max rise slope 104.6 ± 59.6 mV/ms (*n* = 16) to 53.7 ± 30.0 mV/ms (*n* = 10; p = 0.023); rise time 8.4 ± 5.8 ms (*n* = 16) to 13.0 ± 5.6 ms (*n* = 10; p = 0.0.023); rise slope 6.8 ± 6.4 mV/ms (*n* = 16) to 2.6 ± 1.3 mV/ms (*n* = 10; p = 0.035), consistent with reduced availability of fast transient Na^+^ current at a more depolarized baseline (Fig. 3, D and E, and table S1). A smaller proportion of Na_V_1.8^p.R14L^ neurons generated action potentials in response to square-pulse stimulation (WT: 18/27; p.R14L: 12/26), suggesting reduced recruitment under depolarizing inputs. Because dissociated thoracic cultures contain heterogeneous sympathetic subtypes and not all neurons may express Na_V_1.8, these recordings likely underestimate mutation-specific effects within the relevant subpopulation. Collectively, these findings argue against uniform intrinsic hyperexcitability and instead support a depolarizing Na_V_1.8 gain-of-function mutation that shifts membrane potential toward a voltage range in which subthreshold Na^+^ conductances become functionally engaged (*62*–*64*). Under sustained depolarizing input, this depolarization bias may enhance the impact of slow synaptic or cholinergic depolarization on action potential generation, despite reduced availability of fast transient Na_V_ channels. This framework offers a plausible explanation for the exaggerated carbachol-evoked Ca^2+^ responses observed by FLIM and provides a coherent conceptual link between altered somatic spike kinetics in vitro and elevated Na_V_1.8-dependent sympathetic tone in vivo.

### Na_V_1.8^p.R14L^ mice phenocopy excessive sweating

**Fig. 4.**
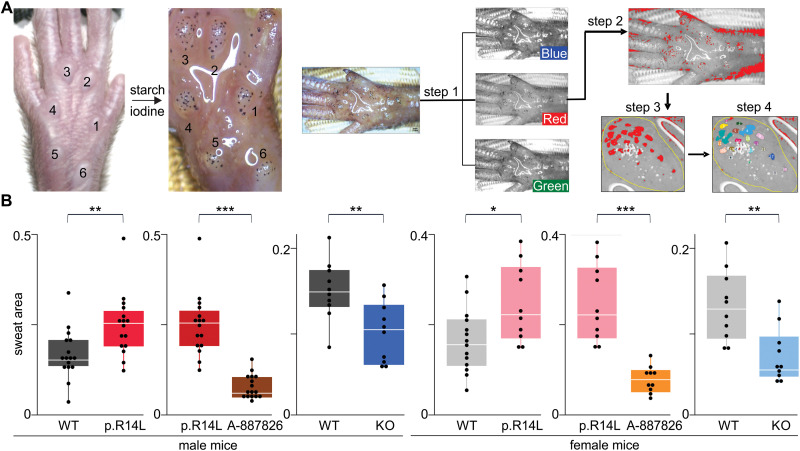
Evaluation of the contribution of Na_V_1.8 to PIH pathophysiology with dynamic sweating measurements. (**A**) Schematic of iodine-starch assay evaluation and analysis. Each hind paw of a mouse has six footpads with sweat glands. Sweat is recognized by the iodine-starch assay as a black-purple dot on the footpad. The sweat area of each footpad was obtained by Image J. The paw surface of the mouse is photographed, and the channels are split (step 1); the grayscale image of the red channel is utilized for subsequent analysis (steps 2–4). By adjusting the threshold, red areas corresponding to sweat droplets on the footpads are selected (step 3), allowing for the definition and calculation of sweat area (step 4). The total sweat area of the six footpads was used for comparative analysis. (**B**) Dynamic sweat measurements, evaluation included comparisons of sweat area between WT (8 male, 7 female) and Na_V_1.8^p.R14L^ (8 male, 5 female), Na_V_1.8^p.R14L^ before and after treatment with 10 mg/kg A-887826 administered i.p. 30 min prior to the test (8 male, 5 female), and WT (*n* = 5 per sex) *vs*. Na_V_1.8^−/−^ mice (*n* = 5 per sex)*.* Two-way ANOVA with specified Holm-Šídák-corrected pairwise comparisons and two-tailed Student’s *t* tests and Welch’s t-test were used. Significant difference with *P < 0.05, **P < 0.01 and ***P < 0.001.

Since eccrine sweat glands in mouse footpads are activated by emotional or anxiety-related stimuli (*65*), mirroring the pattern of palmar and plantar hyperhidrosis in humans, we used the mouse paw as a model for studying PIH-related sympathetic hyperactivity. To preserve physiologic sympathetic tone, we developed a non-sedated, restraint-based adaptation of Minor’s iodine-starch assay (*66*) (fig. S5A). This refinement avoids the suppressive effects of anesthesia on sympathetic output (fig. S5B) and permits dynamic sweat measurement under mild stress conditions, as confirmed by corticosterone levels (fig. S5C). Importantly, although Na_V_1.8 is well known for its role in nociception, the sudomotor phenotypes described here were assessed under non-noxious conditions and therefore reflect baseline sympathetic output rather than pain-evoked stress responses. We optimized assay conditions using 2.5% iodine and 100% starch, with image acquisition at 120 seconds post-application, a time point that captures both the onset and expansion of sweat droplets (fig. S5D). Sweat output was quantified by total sweat area, as we found this metric provides a more accurate and reliable assessment than droplet count due to variability in droplet size within and between mice. Furthermore, sweat area was quantified across six footpads per hind paw (Fig. 4A and fig. S5E), and total sweat function was defined as the summed sweat area across both paws. The assay was robust across sexes and sensitive to pharmacological manipulation, supporting its utility for assessing sympathetic output in vivo.

Na_V_1.8^p.R14L^ mice exhibited significantly increased sweating, 48.9 ± 21.9% (*P* < 0.001) in males (WT: *n* = 8, Na_V_1.8^p.R14L^: *n* = 8) and 50.3 ± 18.7% (p = 0.002) in females (WT: *n* = 7, Na_V_1.8^p.R14L^: *n* = 5), phenocopying the established sex-independent distribution of hyperhidrosis observed in humans (*67*) (Fig. 4B). To test whether this phenotype was Na_V_1.8-dependent, we administered A-887826, a rodent-validated Na_V_1.8-preferential inhibitor with sufficient selectivity for mechanistic in vivo studies when interpreted alongside genetic evidence (*68*), at 10 mg/kg i.p. 30 minutes prior to testing. This treatment reduced sweat production in Na_V_1.8^p.R14L^ mice by 70.3 ± 5.4% (*P* < 0.001) in males (*n* = 8) and 68.6 ± 4.8% (*P* < 0.001) in females (*n* = 5), demonstrating that excessive sweating in mutant mice is reversible with targeted Na_V_1.8 inhibition (Fig. 4B). Consistent with a Na_V_1.8-dependent mechanism, A-887826 also reduced sweat output in WT mice and eliminated the baseline difference between WT and Na_V_1.8^p.R14L^ animals, such that sweat areas converged to comparable levels across genotypes following treatment (fig. S6). Additionally, Na_V_1.8^−/−^ knockout mice showed 33.5% (p = 0.032) and 34.0% (p = 0.002) reductions in sweat output in males and females, respectively (*n* = 5 per sex), further confirming the role of Na_V_1.8 in regulating sweat output (Fig. 4B). The greater suppression of sweating observed with acute pharmacological inhibition compared to the constitutive knockout may reflect compensatory mechanisms during development in Na_V_1.8^−/−^ mice or increased reliance on Na_V_1.8 in a hyperexcitable p.R14L context. These findings underscore the contribution of Na_V_1.8 to PIH pathophysiology and are consistent with sex-independent effects (*2*, *4*).

### The Na_V_1.8^p.C1288W^ loss-of-function variant leads to a reduced sweating phenotype

**Fig. 5.**
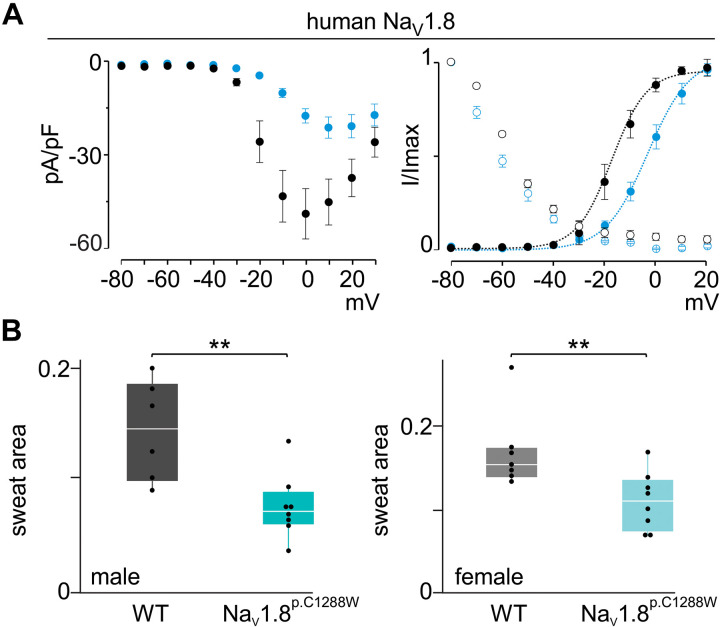
The Na_V_1.8p.^C1288W^ variant exhibits a loss-of-function phenotype and reduces sweating in vivo. (**A**) Electrophysiological characterization of the human Na_V_1.8^p.C1288W^ variant expressed in ND7/23 cells revealed multiple biophysical alterations consistent with reduced channel activity. Compared to wild-type Na_V_1.8 (WT; black), the mutant channel (blue) displayed a pronounced reduction in peak current density (control: *n* = 7, p.C1288W: *n* = 7), and a depolarizing shift in activation voltage (control: *n* = 5, p.C1288W: *n* = 7). These changes indicated partial loss of channel function, which was similarly observed in the corresponding mouse variant. (**B**) In vivo sweat function was assessed using the iodine-starch assay in homozygous Na_V_1.8^p.C1288W^ knock-in mice. Mutant animals exhibited significantly reduced sweat output relative to wild-type littermates, with a 36.7% decrease in males (WT: *n* = 3, Na_V_1.8^p.C1288W^: *n* = 4; *P* < 0.01, two-tailed Student’s *t*-test) and a 35.0% decrease in females (WT: *n* = 4, Na_V_1.8^p.C1288W^: *n* = 4; *P* < 0.01, Mann-Whitney *U* test). One of the four female WT mice was missing data for one paw, resulting in a total of 7 data points.

Prompted by our findings with A-887826 and Na_V_1.8^−/−^ mice, we revisited our WES data to investigate whether loss-of-function variants in Na_V_1.8 were present in individuals with reduced sweating. This analysis identified the Na_V_1.8^p.C1288W^ variant in a patient with anhidrosis. Functional electrophysiological studies demonstrated that Na_V_1.8^p.C1288W^ results in loss of channel function, including a substantial reduction in peak current density (from 52 ± 7 pA/pF to 15 ± 4 pA/pF; *P* < 0.01) and a depolarizing shift in channel activation voltage (V_1/2_ from −16 ± 2 mV, *k* of 7 ± 1, to −3 ± 1 mV, *k* of 8 ± 1; *P* ≤ 0.01) (Fig. 5A). The gating properties of mouse Na_V_1.8^p.C1288W^ were similarly disrupted with a decrease in peak current density from 50 ± 9 pA/pF to 10 ± 4 pA/pF (*P* < 0.01, *n* = 5) (figs. S1 and S7) and a depolarizing shift in channel activation voltage (V_1/2_ from −15 ± 2 mV, *k* of 7 ± 1 to 21 ± 2 mV, *k* of 6 ± 1; *P* ≤ 0.01). To determine whether these biophysical defects translate into physiological consequences, we generated a CRISPR/Cas9-engineered mouse model carrying the Na_V_1.8^p.C1288W^ variant. Strikingly, these mice exhibited a significant reduction in sweat output by 36.7 ± 8.5% in males (WT: *n* = 3, Na_V_1.8^p.C1288W^: *n* = 4; *P* < 0.01) and 35.0 ± 10.6% in females (WT: *n* = 4, Na_V_1.8^p.C1288W^: *n* = 4; *P* < 0.01 compared to wild-type controls) (Fig. 5B), thereby establishing an in vivo link between impaired Na_V_1.8 function and reduced sweating.

**Fig. 6.**
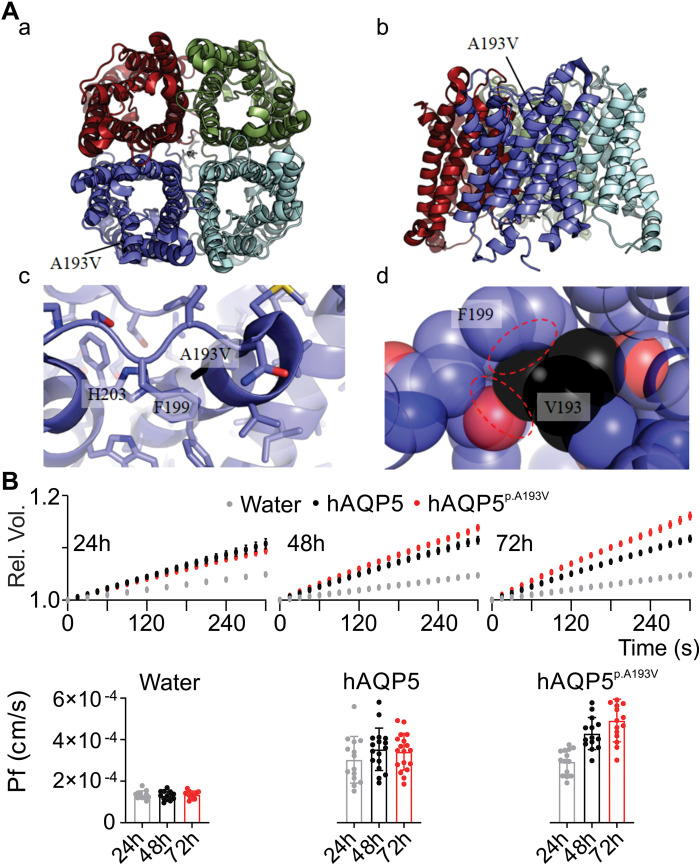
Characterization of the hAQP5^p.A193V^ variant. (**A**) Top (a) and side (b) view of AQP5 (PDB: 3D9S) showing four subunits in different colors. In one subunit, the position of A193 is indicated in black sticks and labeled. (c) Close-up of the environment of A193, indicating that it is buried within a subunit, making contacts with F199 and H203. (d) Space-filling Van der Waals sphere representation showing the mutated V193 in black, causing clashes (red dotted) with F199. This suggests structural perturbations that allosterically may affect water permeation or affect protein stability. (**B**) Upper panel: relative volume increase (V_t_/V_0_) in hypo-osmotic medium (62 mOsm) at 24 hours, 48 hours, or 72 hours post RNA injection. Lower panel: water permeability in cm/s for oocytes injected with nuclease free water (left), hAQP5 WT RNA (middle) or hAQP5^p.A193V^ RNA (right) after 24 hours, 48 hours, or 72 hours post injection. Data is shown as mean ± SD.

The Na_V_1.8^p.C1288W^ variant was inherited by the patient’s son, who reported hyperhidrosis despite the loss-of-function phenotype observed in his mother. This phenotypic discordance suggested the presence of an additional genetic modifier. Targeted analysis revealed no additional candidates, prompting re-evaluation of the full WES dataset. This extended analysis uncovered a de novo variant in *AQP5* (p.A193V) in the son, which was absent from all other sequenced PIH probands. Na_V_1.8 and *AQP5* operate in distinct physiological compartments: Na_V_1.8 in postganglionic sympathetic neurons and AQP5 in eccrine secretory epithelium. We therefore interpret AQP5^p.A193V^ as a gland-level modifier capable of overriding neuronal loss-of-function without implying direct molecular interaction. *AQP5* has previously been implicated in human hyperhidrosis phenotypes (*19*, *33*, *69*–*71*), and although it does not appear to be essential for sweat secretion in mice (*72*), it may play a more prominent role in human eccrine physiology. The p.A193V substitution occurs at a conserved position and is predicted to be ‘likely pathogenic’ according to AlphaMissense (*73*) (pathogenicity score = 0.808), as are most of the amino acid substitutions at this position. Mapping this variant on available crystal structures of human AQP5 shows that A193 is buried within the structure, and that substitution with a larger valine side chain would cause structural clashes with a nearby Phe (Fig. 6A). This could alter protein function, making *AQP5*^p.A193V^ a plausible genetic modifier of the Na_V_1.8^p.C1288W^-associated phenotype. Swelling assays in *Xenopus laevis* oocytes (*74*) substantiated this modifier role (Fig. 6B and table S2). Compared to WT human (h)AQP5, oocytes expressing hAQP5^p.A193V^ displayed accelerated swelling in hypo-osmotic solution (62 mOsm), with a significant increase in permeability (Pf) emerging at 48 hours (p = 0.0267) and becoming highly pronounced at 72 hours (*P* < 0.0001). In contrast, hAQP5 and water-injected controls showed no difference over time. These data indicate that hAQP5^p.A193V^ enhances water permeability, providing a plausible gland-level mechanism capable of counteracting reduced neuronal drive.

### Evaluating therapeutic options for PIH

**Fig. 7.**
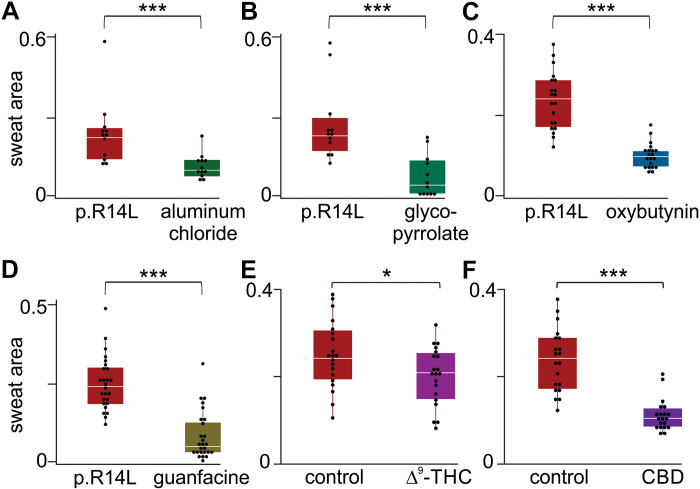
Pharmacological suppression of excessive sweating in Na_V_1.8^p.R14L^ mice. Sweat area was quantified using the iodine-starch assay following treatment with established hyperhidrosis therapies: (**A**) 20% aluminium chloride applied topically once daily for 7 days (treated vs. untreated, *n* = 3 per sex); (**B**) Glycopyrrolate 0.125 mg/kg i.p., administered 30 minutes prior to testing (treated vs. untreated, *n* = 3 per sex); (**C**) Oxybutynin 4 mg/kg i.p., administered 30 minutes prior to testing (treated vs. untreated, *n* = 5 per sex); (**D**) Guanfacine 0.3 mg/kg i.p., administered once daily for 5 days with a final dose 30 minutes prior to testing (treated: *n* = 8 males, 5 females; untreated: *n* = 8 males, 5 females); (**E**) Δ^9^-tetrahydrocannabinol (THC) 2 mg/kg, 30 minutes prior to testing (treated vs. untreated, *n* = 5 per sex); (**F**) Cannabidiol (CBD) 2 mg/kg, 30 minutes prior to testing (treated vs. untreated, *n* = 5 per sex). Statistical comparisons were performed using two-tailed Student’s *t*-tests, Welch’s *t*-tests, or Mann–Whitney *U* tests as appropriate with *P < 0.05, **P < 0.01 and ***P < 0.001.

While our preclinical studies provide insights into the mechanistic basis of hyperhidrosis, translating these findings into effective clinical therapies remains challenging. A-887826 is not available for clinical use. The new Na_V_1.8-inhibiting drug suzetrigine lacks activity on mouse Na_V_1.8 (*38*). Given the genetic heterogeneity of PIH, no single drug is likely to work for all patients. Therefore, we evaluated clinically used treatment strategies in our Na_V_1.8^p.R14L^ mouse model. To systematically assess hyperhidrosis therapies, we applied our iodine-starch assay in Na_V_1.8^p.R14L^ mice, enabling reproducible quantification of sweat function across pharmacologic interventions. We next evaluated four clinically relevant therapies: aluminum chloride, glycopyrrolate, oxybutynin, and guanfacine (*1*, *6*, *7*, *15*, *75*). Aluminum chloride, a first-line topical agent for hyperhidrosis, functions locally by precipitating with mucopolysaccharides to block sweat ducts. Continuous application of 20% aluminum chloride once daily for 7 days resulted in a significant reduction in sweat output in Na_V_1.8^p.R14L^ mice (Fig. 7A). Glycopyrrolate, a muscarinic M3 receptor antagonist which also acts locally, is commonly used as monotherapy and generally has fewer side effects than other anticholinergics. Intraperitoneal administration of 0.125 mg/kg in Na_V_1.8^p.R14L^ mice led to a 72.8 ± 9.9% reduction (*P* < 0.001) in sweat area (Fig. 7B). An observed side effect in mice included urinary retention. Oxybutynin was administered i.p. at 4 mg/kg, producing suppression of sweat output (Fig. 7C). Literature supports its clinical efficacy via local muscarinic receptor antagonism (*7*). Additionally, oxybutynin has been identified in a repurposing screen as a potential Na_V_1.8 inhibitor (*76*), possibly underlying its local anesthetic effects. Guanfacine, a centrally acting α2-adrenoceptor agonist, has previously shown benefit in patients with Na_V_1.7^p.I739V^ variants and comorbid hyperhidrosis (*75*). To assess its relevance to Na_V_1.8-driven phenotypes, Na_V_1.8^p.R14L^ mice were treated with 0.3 mg/kg i.p. guanfacine for five consecutive days, with a final dose given 30 minutes prior to testing. This resulted in a 64.9 ± 6.8% reduction (*P* < 0.001) in sweat output (Fig. 7D). The ability of guanfacine to inhibit Na_V_ channels, including Na_V_1.8 (*75*), provides a possible synergistic mechanistic basis for its effect. Finally, the sweat-inducing effect of pilocarpine, a muscarinic receptor agonist, was confirmed in both control and mutant mice (fig. S8). These experiments establish the Na_V_1.8^p.R14L^ mouse model as a platform for evaluating sweat-suppressing clinical therapies and demonstrate the utility of the modified iodine-starch assay in quantifying pharmacologic modulation of sweat function.

### A possible role for cannabinoids in hyperhidrosis management

A clinically relevant observation emerged from our PIH patient cohort. Multiple individuals reported self-medicating with cannabis, citing notable improvement in sweating symptoms. This anecdotal and reported (*77*) trend prompted mechanistic consideration, as both Δ^9^-tetrahydrocannabinol (THC) and cannabidiol (CBD) have been shown to inhibit Na^+^ currents (*78*–*80*). To evaluate this in vivo, we administered dronabinol (Δ^9^-THC) and CBD in our Na_V_1.8^p.R14L^ mouse model. A single i.p. dose of 2 mg/kg suppressed sweating by 15.7 ± 6.8% (p = 0.038) with THC and 53 ± 5.9% (*P* < 0.001) with CBD (*n* = 5 per sex), compared to untreated Na_V_1.8^p.R14L^ controls (*n* = 5 per sex) (Fig. 7, E and F). These results support the therapeutic potential of cannabinoids in PIH, with the greater efficacy of CBD likely reflecting its stronger activity as a non-selective Na_V_ channel inhibitor (*78*). Together, the findings suggest cannabinoids can mitigate SyNS-driven hyperhidrosis and warrant further investigation into their mechanism of action.

## DISCUSSION

This study identifies a genetically defined subset of PIH as a neurocutaneous ion channelopathy. The Na_V_1.8^p.R14L^ substitution, identified by WES in a therapy-refractory family, produces a gain-of-function channel profile that enhances cholinergic responsiveness in postganglionic sympathetic neurons and drives excessive sweating in vivo (Figs. 1 to 3 and figs. S1 and S2). This phenotype is pharmacologically reversible with Na_V_ channel inhibition (Figs. 4, 5, and 7). Because cholinergic sudomotor neurons constitute only a small fraction of thoracic sympathetic ganglia, we employed a multimodal strategy combining genetics, imaging, electrophysiology, and in vivo phenotyping, to resolve their functional contribution. Together, these data challenge the view of PIH as exclusively gland-intrinsic and provide a mechanistic explanation for failure of topical therapies in neuron-driven cases. They also align with reports linking Na_V_ channel dysfunction to autonomic disorders characterized by altered excitability (*43*, *49*, *81*), in line with the efficacy of thoracic sympathectomy in abolishing hyperhidrosis in selected patients (*28*, *29*), and the broader autonomic symptom burden documented in PIH patients using COMPASS-31 scores (*12*). Consistent with this, questionnaire-based screening revealed features suggestive of neuropathic pain and allodynia, highlighting circuit-dependent expression of *SCN10A* variants.

Although the mechanistic link between Na_V_1.8 dysfunction and sweating is well supported, the modest cohort size limits generalizability and precludes detection of noncoding or structural contributors. Our data further indicate that neural and gland-level mechanisms can interact within individual families. PIH is likely genetically heterogeneous, with variants in other Na_V_ channel subtypes, excitability regulators, or gland proteins such as *AQP5* (*69*–*71*) converging on shared pathways. For example, the Na_V_1.8^p.C1288W^ variant reduced sweating in mice and humans, whereas a co-occurring *AQP5*^p.A193V^ variant in the same human family was associated with hyperhidrosis, consistent with a gland-level modifier effect mediated by increased water permeability (Fig. 6 and fig. S7) (*69*). In this model, AQP5 does not directly regulate Na_V_1.8 but functions as a gland-level modifier capable of overriding neuronal loss-of-function. Similar genotype-phenotype variability has been observed in other Na_V_ channel disorders, including *SCN9A*^p.I1739V^ carriers (*49*), underscoring the complexity of channelopathies shaped by genetic context. More broadly, phenotypic heterogeneity among *SCN10A* variants likely reflects tissue-specific expression across sensory, autonomic, and cardiac circuits, variant-specific biophysical effects, and the influence of genetic modifiers operating in parallel physiological compartments. Together, these factors explain how distinct *SCN10A* variants can manifest as sensory neuropathy, cardiac phenotypes, or PIH without implying a single, uniform disease mechanism.

Clinically, PIH is frequently accompanied by features of sympathetic dysregulation, supporting the possibility that it represents an early or focal manifestation of broader autonomic dysfunction (*8*–*12*). In this context, sweating provides a measurable neurocutaneous readout of systemic autonomic tone. Na_V_1.8 is expressed in DRG, intracardiac neurons, and select central circuits (*37*, *40*, *41*, *54*, *55*). Although our data implicate postganglionic neurons within thoracic sympathetic ganglia as primary effectors, contributions from afferent or central circuits cannot be fully excluded. While Na_V_1.8 is classically associated with nociceptive afferents, the iodine–starch assay was performed without noxious stimulation and under minimal-stress conditions (fig. S5), making a pain-driven stress response unlikely. Moreover, the reciprocal genetic directionality, hyperhidrosis with gain-of-function and hypohidrosis with loss-of-function or knockout, is inconsistent with a purely nociceptive mechanism. Instead, the anatomical specificity and pharmacological reversibility of the phenotype support a dominant role for postganglionic cholinergic sympathetic neurons. The Na_V_1.8^p.R14L^ mouse model therefore recapitulates key physiological aspects of PIH but represents pathogenic potential rather than the full human syndrome. Species-specific differences including sweat gland localization and the absence of thermoregulatory sweating may constrain direct extrapolation. Future validation using hiPSC-derived sympathetic neurons (*82*) from PIH patients will direct assessment of neuron-intrinsic hyperexcitability in a human context. Given that the precise proportion of cholinergic sympathetic neurons in human ganglia remains undefined, our conclusions integrate convergent evidence from genetics, transcript and protein localization, FLIM, electrophysiology, and in vivo sudomotor assays. Therapeutically, these findings support consideration of systemic neuromodulatory strategies in genetically stratified cases rather than exclusively gland-targeted interventions. Although Na_V_1.8 inhibition reversed excessive sweating in mice, translation is complicated by species-dependent pharmacology, as the clinically advanced, human-optimized Na_V_1.8 inhibitor suzetrigine exhibits markedly reduced potency on mouse Na_V_1.8. (*38*). Nonetheless, broader Na_V_ channel-targeting strategies including guanfacine (*75*), oxybutynin (*76*), and cannabinoids showed efficacy in our model, suggesting convergent therapeutic entry points via modulation of sympathetic excitability or glandular responsiveness. Given the heterogeneity of PIH, combination or pathway-targeted approaches may ultimately prove more effective than single-agent therapy. Collectively, our findings establish a genetically defined subset of PIH as a disorder of sympathetic excitability, situating it within the broader spectrum of Na_V_ channelopathies that affect sensory, visceral, and autonomic circuits. Parallels with other dysautonomias, including postural orthostatic tachycardia syndrome, highlight the broader relevance of Na_V_ channel dysfunction to human disease. By identifying a tractable neurocutaneous channelopathy in which neuronal and glandular factors converge on dysregulated sweat output, this work provides a mechanistic basis that supports reclassification of PIH as a neurological condition and for pursuing targeted, stratified therapies.

## MATERIALS AND METHODS

### Participant recruitment, sequencing, and ethics

This study was conducted as part of a Genome-wide Sequencing study entitled, ‘Identify Genes Responsible for Mendelian Disorders’ at Johns Hopkins University (NA_00045758, PI: David Valle). Participants were recruited from inpatient and outpatient clinics, external referrals, and self-registration via a secure web portal. Eligible individuals included those with unexplained Mendelian disorders, locus-heterogeneous phenotypes, or undiagnosed conditions with likely Mendelian inheritance. Written informed consent was obtained from all participants or legal representatives according to institutional guidelines. The study was approved by the Johns Hopkins Medicine Institutional Review Board (NA_00045758), and all procedures adhered to relevant regulations on human subject research and data confidentiality.

### Clinical evaluation of hyperhidrosis and autonomic dysregulation

PIH was defined according to an international consensus statement that patients must have focal, visible, excessive sweating of at least 6 months duration without apparent cause with at least two of the following characteristics: (i) bilateral and relatively symmetric sweating, (ii) sweating that impairs daily activities, (iii) sweating of a frequency of at least one episode per week, (iv) age of onset less than 25 years, (v) there is a positive family history, and (vi) there is cessation of focal sweating during sleep (*1*). Control family members without PIH had no excessive sweating of at least 6 months duration. In addition, the COMPASS-31 (Composite Autonomic Symptom Score-31) questionnaire was used to assess the severity and impact of autonomic dysfunction (*83*). COMPASS-31 is a validated, patient-reported questionnaire with 31 items covering six autonomic domains: orthostatic intolerance, vasomotor, secretomotor (which includes sweating), gastrointestinal, bladder, and pupillomotor symptoms. The HDSS (Hyperhidrosis Disease Severity Scale) questionnaire was used to evaluate sweating (*84*). HDSS is a disease-specific, single-item scale in which patients rate the impact of their sweating on daily activities using a four-point scale with scores of 3 or 4 indicating severe hyperhidrosis and scores of 1 or 2 indicating mild or moderate disease. Neuropathic pain and cutaneous allodynia were assessed using validated, questionnaire-based tools. Neuropathic pain was screened with the Douleur Neuropathique en 4 Questions (DN4) questionnaire (*46*), and cutaneous allodynia with the 12-item Allodynia Symptom Checklist (ASC-12) (*47*).

### Genetic analysis

The WES procedure was previously described by Dhaheri and coworkers (*85*) using genomic DNA captured with the Agilent SureSelect Human All Exon V4 51MB Kit and sequenced on an Illumina HiSeq2000 platform. FASTQ files were aligned to the reference genome (GRCH37) with the Burrows-Wheeler Alignment (BWA 0.5.10) tool. Next, we performed multisample SNV and indel calling on the reduced-read BAM files with GATK’s UnifiedGenotyper. Variant sites were filtered with GATK’s Variant Quality Score Recalibration (GATK version). The annotated files (ANNOVAR) were analyzed using the PhenoDB Variant Analysis Tool (*86*) by selecting the rare (MAF < 0.01) functional (missense, nonsense, stop loss, splice site and indels) heterozygous and homozygous variants in each proband. We excluded variants with a MAF > 0.01 in gnomAD or in our BHCMG sample. Next, each variant and gene were evaluated for their ClinVar, HGMD, OMIM, and mouse phenotype annotations. Finally, we selected for further investigation the gene variants that met the above criteria in heterozygous or homozygous states.

### Animal models

Na_V_1.8^p.R14L^ and Na_V_1.8^p.C1288W^ mice were generated in the Transgenic Core Laboratory at Johns Hopkins University School of Medicine. CRISPR/Cas9 was employed to insert *Scn10a* perturbations into C57BL/6 J mice. The procedure was carried out with the use of CRISPOR, an open-source *in silico* tool that predicts potential off-target effects. All founder lines were sequenced throughout the *Scn10a* gene. Lines that contained the mutation were crossed with wild type C57BL/6 J mice for five generations to reduce as much as possible off target effects. Hereafter, lines that showed the correct genotype were bred to homozygosity and used for further experimentation. Animals were housed in individually ventilated cages (IVC) under standard conditions (12-hour light/dark cycle, 20–25°C, 30–70% humidity) in an IACUC-approved facility, with routine health monitoring. All experiments were conducted using wild-type, Na_V_1.8^p.R14L^, Na_V_1.8^p.C1288W^, and Na_V_1.8^−/−^ mice aged 8–16 weeks. Wild-type C57BL/6 J mice were purchased from The Jackson Laboratory (Bar Harbor, ME, USA). Mice were subsequently bred and maintained in our facility and used for experiments. Genotyping for Na_V_1.8^p.R14L^ and Na_V_1.8^p.C1288W^ was performed via PCR and Sanger sequencing of ear biopsies collected post-weaning. Genomic DNA was extracted from biopsies using the Extracta DNA Prep for PCR (Quantabio, cat. no. 95091) according to the manufacturer’s protocol. AccuStart II PCR Genotyping Kit (Quantabio, cat. no. 95136) was used in PCR amplification. PCR was carried out with the following primers: for Na_V_1.8^p.R14L^, 5ʹ-GGAGCTGGAGACAGAGATAGGGGGC-3′ (forward) and 5ʹ-GCCTTCAAGTCCAACTGAGG-3′ (reverse); for Na_V_1.8^p.C1288W^, 5ʹ-GTGTAAGAAGCACAGAAAGGGACATTTG-3′ (forward) and 5ʹ-GTAGCCCATAGCAACGTTGTCGAAG-3′ (reverse). Amplification was performed in a Veriti thermal cycler (Applied Biosystems, CA, USA) with 40 cycles of 94°C for 20 s, 60°C for 20 s, and 72°C for 50 s (Na_V_1.8^p.R14L^) or 72°C for 40 s (Na_V_1.8^p.C1288W^), with an initial denaturation at 94°C for 5 min and final extension at 72°C for 5 min. PCR products were purified using the QIAquick PCR Purification Kit (Qiagen, cat. no. 28106). Na_V_1.8-Cre knockout (B6.129(Cg)-Scn10a^tm2(cre)Jwo^/TjpJ mice were obtained from The Jackson Laboratory (Bar Harbor, ME, USA). All animal procedures conducted at Johns Hopkins were approved by the Johns Hopkins University Animal Care and Use Committee (protocol number MO24M310). All experiments executed at the University of Gent (UGent) were approved by the ethical committee of the Faculty of Medicine and Health Sciences (ECD 20/80, ECD23/03, ECD23/117). Wild type C57BL/6Rj mice were ordered from Janvier at 6 or 8 weeks old. Homozygous Na_V_1.8^p.R14L^ mice were bred at the UGent animal facility and housed in IVCs in a facility with a 12 h light-dark cycle (20–23°C, 40–70% humidity), with ad libitum access to water and food. Cages were environmentally enriched. Both females and males were used for experimentation between the age of 8–12 weeks. Genotyping was executed as follows: DNA was extracted from the upper toe part of mouse pups (collected at < P7) by incubating the tissue in 50 mM NaOH for 30 minutes at 98°C in the C1000 Touch Thermal Cycler (Bio-Rad). Next, a PCR was run using the forward primer 5ʹ-GG-AGCTGGAGACAGAGATAGGGGGC-3′, reverse primer 5ʹ-GCCT-TCAAGTCCAACTGAGG-3′ and OneTaq DNA Polymerase (New England Biolabs, cat. no. M0480L). The following Thermal cycler protocol was used: 1 min at 94°C, 30 x (30s at 94°C, 30s at 60°C and 1 min 10s at 68°C) and 5 min at 68°C. The samples were Sanger Sequenced (Eurofins) using the forward primer.

### Immunohistochemistry imaging

Sympathetic nerves surgically excised from human subjects were immediately grossly sectioned into 4 mm sections for fixative penetration. A 10% neutral buffered formalin solution was used at room temperature to fix tissues followed by routine dehydration by graded alcohol and xylene. Specimens were then embedded in paraffin blocks. Sections of 5 μm thickness were cut using a microtome, mounted on glass slides. The cellular distribution of *SCN10A* and *SCN5A* proteins in human thoracic tissues was determined using immunofluorescence-based FISH (NA_00045758 PI: Valle). Slices were deparaffinized and rehydrated through three 5-minute xylene baths, two 2-minute 100% ethanol baths, one 2-minute 95% ethanol bath, one 2-minute 70% ethanol bath, and a 1-minute gentle running water rinse. Antigen retrieval was performed using Target Retrieval Solution (Dako, cat. no. S1699, USA), followed by thorough rinsing with deionized water. Non-specific binding was blocked using Endogenous Block Solution (Dako, cat. no. S2003, USA), and slices were incubated with 2.5% normal horse serum (Vector Lab, cat. no. MP-7401) after washing with buffer (Dako, cat. no. S3006). Primary antibodies with human reactivity for *SCN10A* (1:100; NeuroMab, cat. no. 75–166, USA) and *SCN5A* (1:100; Thermo Fisher Scientific cat. no. MA1–27429, USA) were applied and incubated overnight at 4°C. After washing with buffer, slices were incubated with FITC-conjugated secondary antibody Goat anti-mouse IgG (1:500; Thermo Fisher Scientific, cat. no. F-2761, USA) or Goat anti-mouse IgM (1:500; Thermo Fisher Scientific, cat. no. 31992, USA) for 1 hour in the dark. Slides were then washed with buffer and mounted using DAPI-containing mounting medium (Vector Laboratories, cat. no. H-1200, USA) for nuclear counterstaining. Mice were anesthetized and euthanized with 4% volatile isoflurane and subsequently intraperitoneally administered sodium pentobarbital (250 mg/kg). Next, mice were transcardially perfused with PBS (137 mM NaCl, 2.7 mM KCl, 10 mM Na_2_HPO_4_ and 1.8 mM KH_2_PO_4_ adjusted to pH 7.4 with HCl) and subsequently with 4% w/v paraformaldehyde (PFA) solution (Klinipath, cat. no. 4078.9005) until fixation was completed. After tissue isolation in PBS, tissue was postfixed for 45 minutes (sympathetic ganglia and DRG) or overnight (brains) at 4°C. Mouse tissues were cryoprotected at 4°C by sequential incubation in 15% and 30% sucrose solutions (in PBS). For freezing, sympathetic ganglia and DRG were embedded in freezing medium (ImmunoLogic, cat. no. 1620-C) and placed directly at −80°C, while brain tissue was snap-frozen in isopentane (VWR, cat. no. 24872.298) chilled with liquid nitrogen. All samples were stored at −80°C until further processing. Brain tissue was transferred to −20°C the evening prior to cryosectioning. DRG and sympathetic ganglia were sectioned directly from −80°C storage. Cryosections (15 μm) were cut on a Leica CM1950 cryostat and mounted onto Superfrost Plus Adhesion slides (Epredia, cat. no. J7850AMNZ), then stored at −80°C. On the day of use, slides were removed from the freezer, and regions of interest were delineated using a hydrophobic barrier PAP pen (Vector Laboratories, cat. no. H-4000). Slides were air-dried for 1 hour and 15 min at room temperature. Sections were washed twice in PBS (5 min each), then sequentially incubated in 0.5% and 1% hydrogen peroxide (H_2_O_2_ in PBS; Sigma-Aldrich, cat. no. H1009) for 30 and 60 min, respectively, to quench endogenous peroxidase activity. Following two additional PBS washes (5 min each), tissue was blocked for 45 min in blocking buffer (0.4% fish skin gelatin [FSG; Sigma-Aldrich, cat. no. G7765] and 0.2% Triton X-100 [Sigma–Aldrich, cat. no. X100] in PBS). Overnight incubation at 4°C was performed with the following primary antibodies diluted in blocking buffer: rabbit anti-Na_V_1.8 (1:500; Alomone Labs, cat. no. ASC-016) and chicken anti-tyrosine hydroxylase (TH) (1:500; Abcam, cat. no. ab76442). The next day, sections were washed three times (10 min each) in blocking buffer and incubated for 1 hour at room temperature in the dark with the secondary antibodies: goat anti-rabbit IgG Alexa Fluor 647 (1:500; ThermoFisher, cat. no. A-21245) and goat anti-chicken IgY Alexa Fluor 488 (1:500; ThermoFisher, cat. no. A-11039). After three PBS washes (5 min each), nuclei were counterstained with DAPI (1 μg/mL in PBS; Merck Life Science, cat. no. D9542) for 5 min, followed by two additional PBS washes. Slides were mounted using ProLong Gold Antifade Mountant (ThermoFisher, cat. no. P36930) and cover slipped. Confocal imaging was performed on a Leica TCS SP8 X using a 25×/0.95 NA water-immersion objective. Acquisition parameters: scan speed 600 Hz, pinhole 1 AU, zoom 1, line averaging 6. For DAPI, gain 11% and laser intensity 1% were used; for Alexa Fluor 488 and 647, gain 30% and laser intensity 4%.

### Whole mount imaging

Sympathetic ganglia were blocked (5% FSG and 0.2% Triton X-100 diluted in PBS) overnight and thereafter stained for 3 days with chicken anti-TH (1:50). Then, the tissue was washed 3 times for 15 minutes and additionally six times for one hour with PBS. Next, ganglia were incubated with goat anti-chicken IgY AF488 (1:200) for 2 days. To remove unbound antibodies, the tissue was washed 3 times (15 minutes) and additionally 6 times (1 hour) with PBS. Finally, the tissue was mounted with Vectashield Antifade Mounting medium with DAPI (Vector laboratories, cat. no. H-1200-10). Steps were carried out in 96-well plates on a shaker at room temperature and tissue was transferred to a new well using forceps. A Nikon eclipse Ti epifluorescence microscope (Melville, NY, USA) with a 10x/0.3 air objective and a FITC filter were used for imaging.

### RNA in situ hybridization

Sympathetic ganglia were freshly isolated in ice-cold Ringer solution (140 mM NaCl, 5 mM KCl, 10 mM HEPES, 10 mM glucose, 2 mM MgCl_2_ and 2 mM CaCl_2_, pH 7.4) and fixed for 2–3 hours with 4% PFA at 4°C. After 3 washing steps with PBS, the tissue was stored in PBS at 4°C until processing. Tissue was processed using the following protocol on the Leica HistoCore PEARL tissue processor: 10 min 70% ethanol, 10 min 80% ethanol, 10 minutes 95% ethanol, 3x10 min 100% ethanol, 3x10 min xylene, 3x10 min paraffin. All steps were carried out at 45°C except the paraffin step (65°C). Finally, the tissue was paraffin embedded with the Leica HistoCore Arcadia embedding station. After paraffin embedding, sections of 5 μm were made employing the Leica HistoCore Multicut R microtome. All equipment and the surrounding bench were cleaned with RNaseZap (Sigma-Aldrich, cat. no. R2020) to avoid RNA degradation. The sections were mounted on microscopy slides by placing them onto a droplet of nuclease-free water (Sigma-Aldrich, cat. no. W4502) and placing the slide on a heated plate at 46°C. After the sections had flattened, the slide was held vertically, and the water was removed by gently flicking the slide. Sections were dried overnight at 37°C. The RNAscope Multiplex Fluorescent v2 Assay (Advanced Cell Diagnostics, cat no. 323100, Newark, CA, USA) was used according to manufacturer’s instructions with standard pretreatment conditions (15 min. Target Retrieval and 30 min. Protease Plus). The negative control probe used was *B. subtilis* 3-plex (Advanced Cell Diagnostics, cat no. 320871). The positive control used was *M. musculus* 3-plex for housekeeping genes (cat no. 320881), and the tested targets included *M. musculus* ChaT (Advanced Cell Diagnostics, cat no. 408731-C2) and *SCN10a* (Advanced Cell Diagnostics, cat no. 426011). For immunostaining after ISH, sections were blocked during 30 min. With 0.5% IgG-free BSA (Jackson ImmunoResearch, Newmarket, UK) in PBS + 0.01% Tween-20. Primary antibody (rabbit anti-Na_V_1.8, 1:350) was incubated overnight at 4°C and detected using donkey anti-rabbit IgG DyLight 755 (ThermoFisher Scientific, cat. no. SA5–10043, Waltham, MA, USA) diluted at 1:200. Imaging was performed with an Axio Scan.Z1 high throughput slide scanner (Zeiss, Jena, Germany) at 20x magnification. RNA in situ hybridization, immunostaining and imaging was performed by the VSTA core facility at VUB (https://vsta.research.vub.be). Of note: Na_V_1.8 channels are trafficked from the soma into axons and presynaptic terminals and exhibit a relatively long membrane residence time. Quantitative studies in sensory neurons indicate that the plasma-membrane pool of Na_V_1.8 has a half-life of approximately 50 hours, with an intracellular pool of about 30 hours (*87*). In contrast, neuronal mRNAs typically display much shorter half-lives, often less than 6 hours,and are largely restricted to somata (*88*). Because RNAscope detects somatic transcripts, whereas IHC visualizes both somatic and axonal or varicose protein distribution, IHC typically yields more extensive labeling. This pattern is expected in sympathetic ganglia, particularly given the rarity of cholinergic sympathetic neurons. Accordingly, we interpreted Na_V_1.8 localization patterns using convergent evidence across IHC, RNAscope, FLIM, and in vivo sudomotor functional assays.

### Primary mouse sympathetic neuron culture

Coverslips were acid washed for 3 days in 1 M HCl. Next, they were washed 3 times with water and stored in 70% ethanol at 4°C until use. Coverslips were coated within 2 weeks of use with 0.05 mg/ml Poly-D-Lysine (Gibco, cat. no. A38904–01) diluted in PBS (Gibco, cat. no. 10010–023) during one hour at room temperature (storage at 4°C). Coverslips were coated freshly with 0.01–0.02 mg/ml laminin (Sigma-Aldrich, cat. no. L2020) dissolved in PBS. Mice were euthanized with cervical dislocation. Thoracic sympathetic paravertebral ganglia were isolated from the surrounding tissue in ice cold artificial cerebrospinal fluid (119 mM NaCl, 26.2 mM NaHCO_3_, 2.5 mM KCl, 1 mM NaH_2_PO_4_, 1.3 mM MgCl_2_, 10 mM glucose) which was continuously bubbled with carbogen (5% CO_2_, 95% O_2_). Cleaning of the ganglia was executed in Hanks’ balanced salt solution (HBSS; Gibco, cat. no. 14185–052) which was supplemented with 10 mM HEPES. Attached fat was removed, and the chain was cut into individual ganglia. The individual ganglia were submerged in enzyme solution composed of 2.5 mg/ml collagenase type 2 (Worthington-Biochem, LS004176, 270 u/mg) and 2.5 mg/ml dispase (Gibco, cat. no. 17105–041, 1.82 u/mg), dissolved in HBSS. The tube was placed in a heat block (37°C) for 40 minutes and manually rocked back and forth every 5 minutes to allow optimal access of the enzyme to the tissue, and to prevent the ganglia from sticking to each other. The ganglia were washed 3 times with DMEM/F12 with GlutaMAX (Gibco, cat. no. 31331–028) (supplemented with 10% fetal bovine serum (Sigma-Aldrich, cat. no. F4135) and 1% Penicillin-Streptomycin (Pen-Strep; Gibco, 15140–122)) to allow serum-mediated inactivation of the enzymes. Finally, the ganglia were washed once with neurobasal plus medium (Gibco, A35829–01) (supplemented with 2% B-27 Plus supplement (Gibco, A35828–01), 0.5 mM GlutaMAX-I (Gibco, 35050–038) and 1% Pen-Strep). Mechanical trituration of the tissue was performed by pipetting the ganglia up and down with glass Pasteur pipettes with decreasing diameter until most of the tissue had been broken down into smaller fragments. Cells were centrifuged for 2 minutes at 100 g, and the upper part of the supernatant was removed so that enough volume was left to place 15 μl of cell suspension on each coated coverslip. After removal of the laminin, the cells were plated and placed in an incubator (37°C, 5% CO_2_). Following 40 minutes incubation, neurobasal plus medium with additionally supplemented mouse beta-NGF protein (50 ng/ml for voltage-clamp (VC) and 25 ng/ml for current-clamp (CC); Bio-techne, cat. no. 1156-NG) and mouse GDNF protein (50 ng/ml for VC and 2 ng/ml for CC; PeproTech, cat. no. 450–44) was added to fill the cell culture wells. Cells were used for VC and FLIM experiments between 18 and 25 hours after dissociation, and between 6–8 days for CC. The time of euthanasia until addition of final medium was ±3.5 hours.

### Fluorescence lifetime imaging microscopy

The stock solution of Oregon Green BAPTA-1 Acetoxymethyl Ester (OGB-1-AM, 1 mM DMSO, Invitrogen, cat no. O6807) was diluted in Neurobasal plus medium to a final concentration of 2.5 μM and incubated with mouse primary sympathetic neurons (T1-T12; 37°C, 5% CO_2_, 200 μl per well) for 1 hour prior to imaging. Live imaging experiments were conducted in Ringer solution with a 5 mM starting concentration of KCl. Cells were stimulated at three different times with 10 mM KCl, 100 μM carbachol and in the end with 75 μM KCl to raise the Ca^2+^ levels maximally, added from 10X concentrated stocks, and homogeneous distribution was assured through manual resuspension while imaging. Confocal FLIM microscopy was performed on a Stellaris 8 Falcon microscope, (Leica Microsystems, Ghent Light Microscopy Core, UGent), equipped with a white light laser (440–790 nm), HC PL Apo 40×/1.25 GLYC corr objective, HyD X and trans PMT detectors (brightfield images), temperature-/CO_2_-controlled incubator, and dedicated LAS X acquisition and analysis software (ver. 4.6.0) (*89*). A 40×/1.25 GLYC corr. Objective was used, with typical settings: 80 MHz white-light laser frequency pulse, scan speed 600 Hz, pixel dwell time 1.725 μs, pinhole 2.5 airy units, 1- line repetition rate, 512 by 512 pixels resolution. The following general excitation and emission settings were used for fluorescence imaging: 488 nm excitation line (4–8%), detection window 498–710 nm. Regions of interest (ROIs) were defined as single-cell somatic regions with unambiguous boundaries, selected using LAS_X magic wand segmentation, an interactive, region-growing tool used to segment and define ROIs based on pixel values. ROIs containing overlapping cells/debris were excluded. Only cells showing a robust response to the final 75 mM KCl challenge were included as a viability control. Pixel binning (=3) was applied within each ROI to improve signal-to-noise ratio by aggregating photons from neighboring pixels, facilitating more reliable lifetime measurements while maintaining an appropriate balance between spatial resolution and signal sensitivity. To analyze the fluorescence decay kinetics, we employed global fitting to assess the goodness of fit across all ROIs. The decay curves showed an expected bi-exponential decay, with component lifetimes in close agreement with values reported in the literature (*90*), confirming the accuracy of the calcium dynamics measurements. Lifetime traces and fluorescence lifetime decays were then extracted for each ROI as .csv files. Brightfield and fluorescence intensity images, along with fluorescence lifetime timelapse tracking calcium dynamics, were exported from LAS X software and are provided in Supplementary Materials. FLIM timelapse sequences were visualized using the “Turbo” lookup table to enhance contrast and help visualize lifetime changes. Data presentation and statistical analysis were conducted using a custom R script developed in RStudio including mean lifetime time traces for comparison of groups, single-cell lifetime plots, boxplots at key time points, and time traces comparing intensity and lifetime signal to comprehensively illustrate the experimental results. Each trace in the single-cell lifetime graphs represents Ca^2+^ dynamics over time for individual cells. To supplement this graph, boxplots are provided to visualize the distribution of fluorescence lifetimes across the different groups. Statistical analysis was performed to compare fluorescence lifetimes between groups across the defined temporal phases.

### Electrophysiological assays

Pipettes were pulled using a P-1000 puller (Sutter Instruments, USA) and capillary glass with microfilament (A-M systems, cat. no. 596700, USA) with a resistance between 2–4 MOhm. Data were acquired using a Multiclamp 700B amplifier (Moleculare devices, USA), Digidata 1140A digitizer (Molecular devices, USA) and pClamp 10 software (Moleculare devices, USA). Series resistance for all cells was corrected electronically up to 80%. The -P/4 protocol was used to subtract the linear leak and capacitive transients in VC mode. Data were acquired at a sampling rate of 20 kHz and filtered at 10 kHz. An Ag-AgCl pellet in the bath (VC) or in a separate chamber (CC) in combination with an agar bridge (2% agarose in 3 M KCl) was used as reference electrode. For VC recordings, the bath solution consisted of (in mM): 140 NaCl, 3 KCl, 10 HEPES, 20 TEA-Cl, 1 CaCl_2_, 2 MgCl_2_, 0.05 CdCl_2_ and 500 nM tetrodotoxin citrate (TTX; Tocris, cat. no. 1069; 315 mOsm). The pipet solution consisted of (in mM): 110 CsCl, 20 CsF, 2 EGTA, 10 HEPES, 10 NaCl, 4 MgATP (Biosynth, cat. no. NA44288) and 0.4 Na_2_GTP (Sigma-Aldrich, cat. no. G8877; 305 mOsm). For CC recordings, the bath solution consisted of (in mM): 140 NaCl, 3 KCl, 10 HEPES, 2 CaCl_2_ and 2 MgCl_2_ (310 mOsm). The pipet solution consisted of (in mM): 140 KCl, 0.5 EGTA, 5 HEPES and 3 MgATP (295–300 mOsm). Osmolarity was corrected with glucose and pH was respectively adjusted to 7.4 with NaOH (bath solution), CsOH (pipet solution in VC), and KOH (pipet solution in CC). After obtaining the whole-cell configuration, a 2–3-minute wait was introduced to allow the exchange of the pipet solution with the cell inside and to assess the stability of the seal. Holding potential for all VC protocols was −90 mV. Between each sweep, a waiting interval of 5 s was employed. All recordings (VC and CC) were done at 22°C. The current-voltage (IV) and conductance-voltage (G-V) protocol consisted of a 100 ms pulse with increasing voltage steps of 5 mV from −90 mV to 40 mV. The steady-state inactivation (SSI) or channel availability protocol consisted of a 500 ms prepulse with increasing voltage steps of −90 mV until 40 mV in steps of 10 mV, followed by a 100 ms pulse to −10 mV. Peak currents were measured during the 100 ms pulse. For the IV and GV protocols, peak currents at each voltage were normalized to cell capacitance to obtain current density (pA/pF) which was plotted against voltage. In addition, the conductance (G) was calculated using the equation G = I/(V-E_rev_). G values were normalized to the maximum G value to obtain the G-V curve. For the SSI protocol, peak currents at each voltage were normalized to the maximum peak value and plotted against voltage. Where appropriate, curves were fitted using a standard Boltzmann function. Passive membrane properties were assessed using 200 ms hyperpolarizing current steps from −25 to −45 pA in −5 pA increments. Input resistance (R_in_) was calculated from the steady-state voltage deflection (ΔV/I). The membrane time constant (τ) was obtained by single-exponential fitting of the voltage response, and membrane capacitance (Cm) was calculated as τ/R_in_. Resting membrane potential was determined from the 200 ms baseline preceding current injection. Passive membrane parameters were averaged across the six hyperpolarizing steps for analysis. Both firing and non-firing neurons were included. Cells with R_in_ < 185 MΩ or exhibiting spontaneous action potentials were excluded.

Action potential properties and rheobase were determined using 500 ms depolarizing current steps (2 pA increments). Cells that did not fire at ≤60 pA were subsequently tested in 25 pA increments up to 575 pA. Cells that failed to generate an action potential were classified as non-responsive. Action potential parameters were extracted in Clampfit (Molecular Devices, USA) from the spike elicited at rheobase. Threshold was defined as the membrane potential at which dVm/dt first exceeded 20 mV/ms. The junction potential (6 mV for VC, 4.5 mV for CC) was calculated using the Junction potential calculator in Clampex (Molecular devices, USA). Similar VC protocols were used for ND7/23 cells, purchased from Sigma-Aldrich and cultured in Dulbecco’s modified Eagle’s medium (DMEM; Gibco), supplemented with 10% fetal bovine serum (Gibco), 1% penicillin-streptomycin (Gibco), and 2 mM l-glutamine (Gibco). Cells were kept in an incubator at 37°C and 5% CO_2_ and periodically screened for mycoplasma contamination. Cells were plated the day before transfection and used at a confluence of ~70%. Transfection in a 24-well plate with 500 ng of human or mouse Na_V_1.8 cDNA, or their variants, in the pCMV6-Entry vector (Origene, USA) together with 150 ng mCherry was performed using PolyJet (SignaGen), according to the manufacturer’s protocol and with a DNA (micrograms)–to–PolyJet reagent (microliters) ratio of 3:1. Electrophysiological recordings were carried out 24-48 hours post-transfection. The extracellular solution contained (in mM): 145 NaCl, 4 KCl, 1.8 CaCl_2_, 1 MgCl_2_, 10 HEPES, 1 μM TTX. pH 7.36, 305 mOsm. The intracellular solution contained (in mM): 110 CsF, 10 CsCl, 10 TEACl, 2 EGTA, 10 HEPES, 10 NaCl pH 7.36, 300 mOsm. Osmolarity and pH were adjusted with 1 M Glucose and NaOH or CsOH respectively.

### Swelling assay in *X. laevis* oocytes

The cDNA sequences of hAQP5 and hAQP5^p.A193V^ (in pcDNA3.1; Genscript, USA) were confirmed by automated Sanger sequencing (Eurofins Genomics, Germany). RNA was synthesized using T7 polymerase (mMessage mMachine kit, Invitrogen, USA) after linearizing the cDNA with an appropriate restriction enzyme (New England Biolabs, USA). RNA was microinjected at 300 ng/μl into defolliculated *X. laevis* oocytes (Nasco, USA) and incubated for 24–72 hours at 17°C in Barth’s medium (in mM): 96 NaCl, 2 KCl, 5 HEPES, 1 MgCl2, and 1.8 CaCl2 supplemented with 50 μg/ml gentamycin, pH 7.4 with NaOH, 206 mOsm (Sigma, USA)). Nuclease-free water-injected oocytes were used as control. The use of frogs complied with national and Flemish guidelines adhered to by the UGent University Animal Care and Use Committee. Swelling assays (*74*) were performed at 24 hours, 48 hours, and 72 hours after injection by incubating individual oocytes in a 96-well plate in hypo-osmotic Barth’s medium (62 mOsm). Brightfield images (2944 x 2175 μm; 1224 x 904 pixels; 16 bit) were taken every 15 s for up to 15 min with the Lionheart FX automated microscope (2.74X/0.12NA Zeiss Plan Fluorite objective) using Gen5 software (Agilent Technologies, USA). Image data analysis was performed using Fiji (ImageJ). Briefly, binary images were made by automatic thresholding (default) 8-bit images after which the 2D oocyte surface area (A_2D_) was measured. Subsequently, their volume was calculated according to *V* = (4/3)π*r*3 where *r* = √(*A*_2*D*_/π). Oocyte water permeability (Pf) was calculated according to *Pf*= 1/(*A*_3*D*_ ∗ *Vw* ∗ ∆*osm*) ∗ *dV*/*dt* where A_3D_ is the 3D oocyte surface area (m^2^), VW is the partial molar volume of water (18e-6 m^3^/mol), Δosm is the osmotic gradient (mol/m^3^) and dV/dt is the initial slope of volume increase during the first 60s (m^3^/s). Statistical analysis was done in GraphPad Prism 10.6.1 using a one-way ANOVA test with post hoc Tukey correction for multiple testing.

### Iodine-starch assay

The adapted iodine-starch assay was conducted using wild-type, Na_V_1.8^p.R14L^, and Na_V_1.8^p.C1288W^, Na_V_1.8^−/−^ mice 8–16 weeks old at room temperature (22°C). The soles of the hind paws were painted with a 2.5% (w/v) iodine solution (Sigma-Aldrich, cat. no. 207772) in ethanol and allowed to dry completely. The same surfaces were then coated with a starch solution consisting of 1 mg/mL starch (Sigma-Aldrich, cat. no. 33615) in castor oil (Sigma-Aldrich, cat. no. 259853). Two minutes after application of the starch solution, images of the entire sole were acquired using a stereo microscope (AmScope, SM-3 T) and a microscope camera with software (AmScope, AF202). Each mouse was tested on both hind paw soles. Images were analyzed using ImageJ software (version 1.54 g, National Institutes of Health, Bethesda, MD, USA). The sweat area was quantified by measuring the total area of black dots present on the footpads. Each hind paw contains six footpads. The total sweat area for each paw was determined by summing the area of all six footpads on either the left or right hind paw. The following drugs and administration protocols were applied under identical conditions across all experimental groups to ensure pharmacological comparability using the iodine-starch assay: pilocarpine (2.5 mg/kg; Sigma-Aldrich, cat. no. PHR1493), glycopyrrolate (0.125 mg/kg; Sigma-Aldrich, cat. no. SML0029), and oxybutynin (4 mg/kg; Sigma-Aldrich, cat. no. O2881). Drugs were dissolved in PBS and administered intraperitoneally at a volume of 10 mL/kg, 30 minutes prior to the assay. Guanfacine (0.3 mg/kg; Sigma-Aldrich, cat. no. G1043) in PBS was administered intraperitoneally at 10 mL/kg once daily for five consecutive days prior to the assay, and again 30 minutes before testing. A-887826 (10 mg/kg; Sigma-Aldrich, cat. no. SML0345) was prepared in a vehicle consisting of DMSO, Tween 80, and PEG 300, and administered intraperitoneally at 10 mL/kg, 30 minutes before the assay. Cannabidiol (2 mg/kg; Sigma-Aldrich, cat. no. 1089149) and Δ^9^-tetrahydrocannabinol (2 mg/kg; Sigma-Aldrich, cat. no. 1651621) were dissolved in a mixture of DMSO, Tween 80, and saline, and administered intraperitoneally at 10 mL/kg, 30 minutes before testing. Aluminum chloride (20%; Sigma-Aldrich, cat. no. 229393) was applied topically to the paw soles using a brush twice daily for 7 consecutive days prior to the assay. To examine sweating under sedation, mice were anesthetized using 3% isoflurane (Vet One, cat. no. 13985–528-60) in 100% oxygen at 1 L/min (induction), maintained at 1%, or with an intraperitoneal injection of ketamine (87.5 mg/kg; Vet One, cat. no. 13985–584-10) plus xylazine (12.5 mg/kg; Dechra, cat. no. 17033–099-05). Anesthesia depth was confirmed using physiological indicators such as respiratory and heart rate, and by testing for the absence of reflexes (e.g., tail pinch). Mice were transferred to a restraint device for assay once an appropriate depth of anesthesia was verified.

### Plasma corticosterone immunoassay

Mice were placed in a restraint device and underwent the iodine-starch assay on the left and right hind paws. As a control, 50 μl whole blood was collected from the jugular vein before placing each mouse in the restraint device without anesthesia. After the assay, mice were released from the restraint, and whole blood was immediately drawn into an EDTA-anticoagulated blood collection tube in the same manner without anesthesia. The collected blood samples were centrifuged at 3000 rpm for 15 min at 4°C. The plasma samples were collected and frozen at −20°C until analysis of corticosterone levels. Corticosterone in plasma samples (final dilution, 1:100) was measured by a competitive immunoassay (Corticosterone Competitive ELISA kit, Invitrogen, USA). The plate was read immediately at 450 nm on an ELISA plate reader (iMark Microplate Absorbance Reader, Bio-Rad, USA).
