# Supplementary material for: A neurocutaneous NaV1.8 channelopathy underlies a genetic subtype of primary idiopathic hyperhidrosis
Source: Sci Adv. 2026 Jul 17;12(29):eaed3221. doi: 10.1126/sciadv.aed3221 (PMC13378580; doi:10.1126/sciadv.aed3221)
Supplement: Supplementary file 1 — Figs. S1 to S8 Tables S1 and S2 Legends for movies S1 and S2 [file sciadv.aed3221_sm.pdf]

Science Advances
AAAS

Supplementary Materials for

A neurocutaneous $Na_V1.8$ channelopathy underlies a genetic subtype of primary idiopathic hyperhidrosis

Suguru Yamauchi *et al.*

Corresponding author: Frank Bosmans, frank.bosmans@vub.be; Malcolm V Brock, mabrock@jhmi.edu

Sci. Adv. **12**, eaed3221 (2026)
DOI: 10.1126/sciadv.aed3221

The PDF file includes:

Figs. S1 to S8
Tables S1 and S2
Legends for movies S1 and S2

Other Supplementary Material for this manuscript includes the following:

Movies S1 and S2

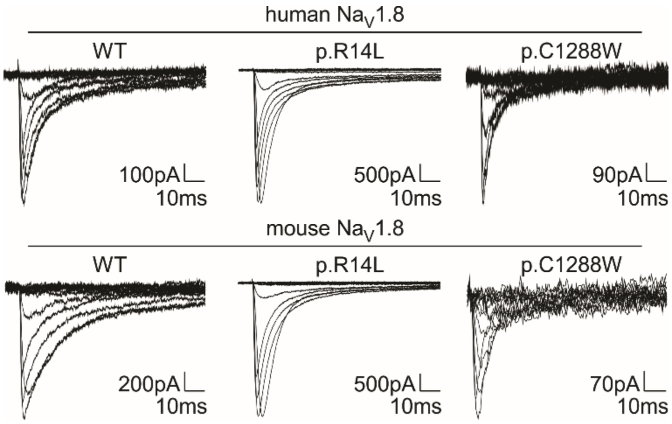

Figure S1: Representative whole-cell $Na_V1.8$ current traces recorded in ND7/23 cells. Example voltage-clamp traces are shown for wild-type (WT) human $Na_V1.8$, WT mouse $Na_V1.8$, and the p.R14L and p.C1288W variants. Currents were recorded from ND7/23 cells held at −90 mV and evoked by 200 ms depolarizing voltage steps applied in 10 mV increments. Axes are indicated in the figure.

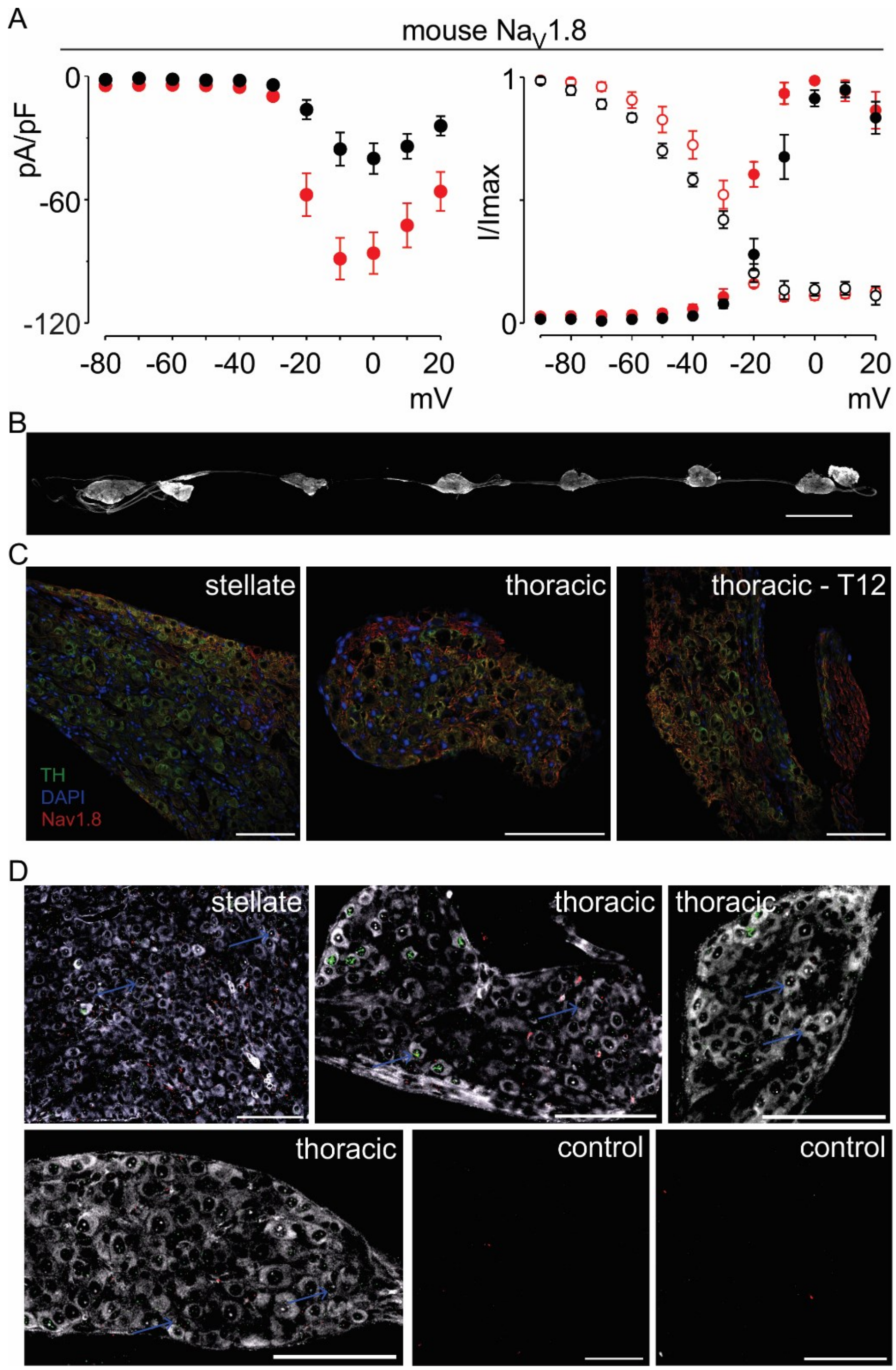

Figure S2: Na_V1.8 function and expression in mouse sympathetic neurons. (A) Whole-cell voltage-clamp analysis of mouse Nav1.8 wild-type (black) and p.R14L mutant (red) channels expressed in ND7/23 cells. As in the human orthologue, the p.R14L mutation nearly doubles peak current density (control: n = 8, p.R14L: n = 8 cells) and induces a hyperpolarizing shift in the voltage-dependence of activation (control: n = 5, p.R14L: n = 8 cells), consistent with a gain-of-function phenotype. **(B)** Whole mount staining of part of the thoracic sympathetic chain show robust expression of TH (scale bar 1 mm) **(C)** Staining performed on cryosections (15 µm thick) of the stellate ganglion (left), a thoracic ganglion between T2-12 (middle), and the T12 thoracic

ganglion (right) revealed expression of Nav1.8 (red) and TH (green) with yellow indicating overlap. DAPI staining is shown in blue (scale bar = 100 μm). **(D)** RNA *in situ* hybridization detection of Nav1.8 (green) and ChaT (red) transcripts in Nav1.8 immunoreactive stellate and T2-T12 thoracic ganglion cells (grey). Blue arrows in the left panel indicate examples of cells where both Nav1.8 and CHAT transcripts are present. As a negative control, both secondary antibody only and negative control probes were used (scale bar = 100 μm). Nav1.8 protein extends into axons and terminals and has a longer half-life than mRNA, whereas RNAscope detects somatic transcripts only. Stronger and more widespread IHC labeling therefore reflects protein trafficking and stability rather than probe sensitivity differences. The thoracic panel shows a wider field of the same preparation presented at higher magnification in Fig. 2D.

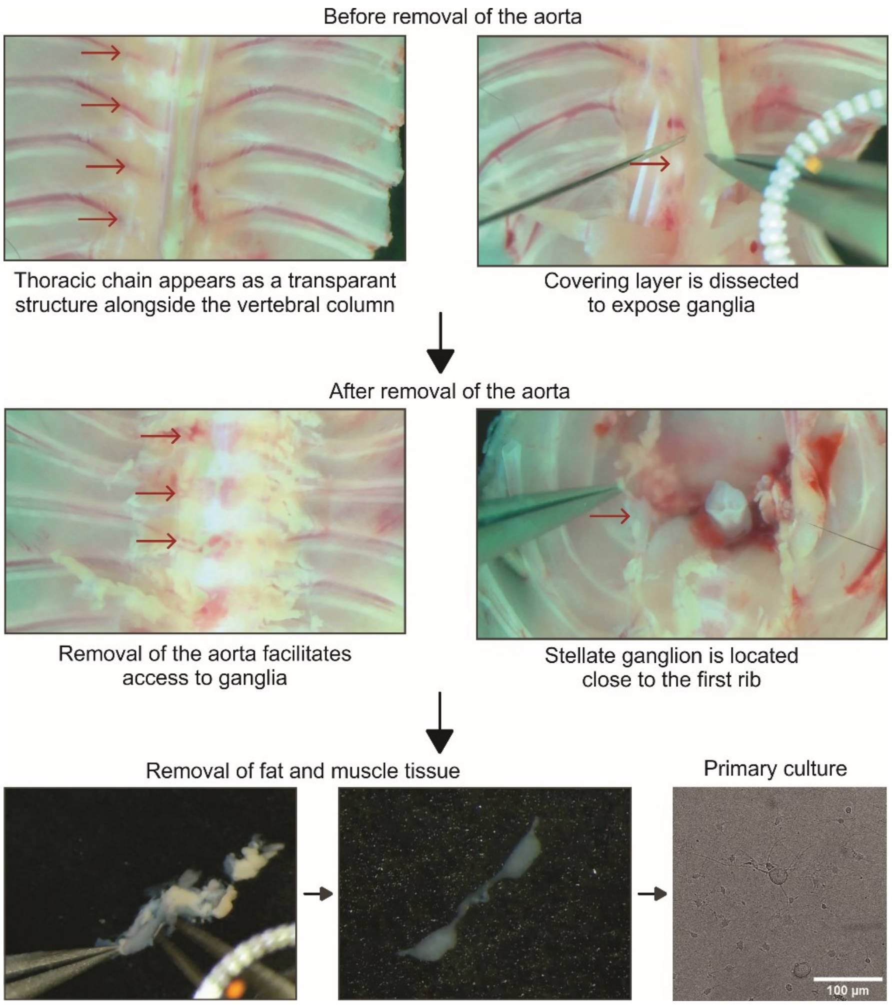

Figure S3: Dissection of the paravertebral sympathetic chain from mice. The thoracic sympathetic chain runs alongside the vertebral column anterior to the heads of the ribs and is covered by a thin layer of parietal pleura. Due to its translucent nature the chain is initially poorly visible (ganglia and interganglionic nerve are indicated by red arrows). After removal of the aorta and the parietal pleura, the sympathetic chain becomes readily apparent. The chain is resected *en bloc* by sharp dissection together with the contiguous underlying muscle and adipose tissue, ensuring removal as a single, anatomical specimen. Retention of the associated muscle and adipose tissue is critical for maximizing the efficiency of the enzymatic dissociation process used in subsequent primary culturing of ganglionic cells. The lower right panel shows a thoracic sympathetic culture, 24 hours post-dissociation. Scale bar is 100 µm.

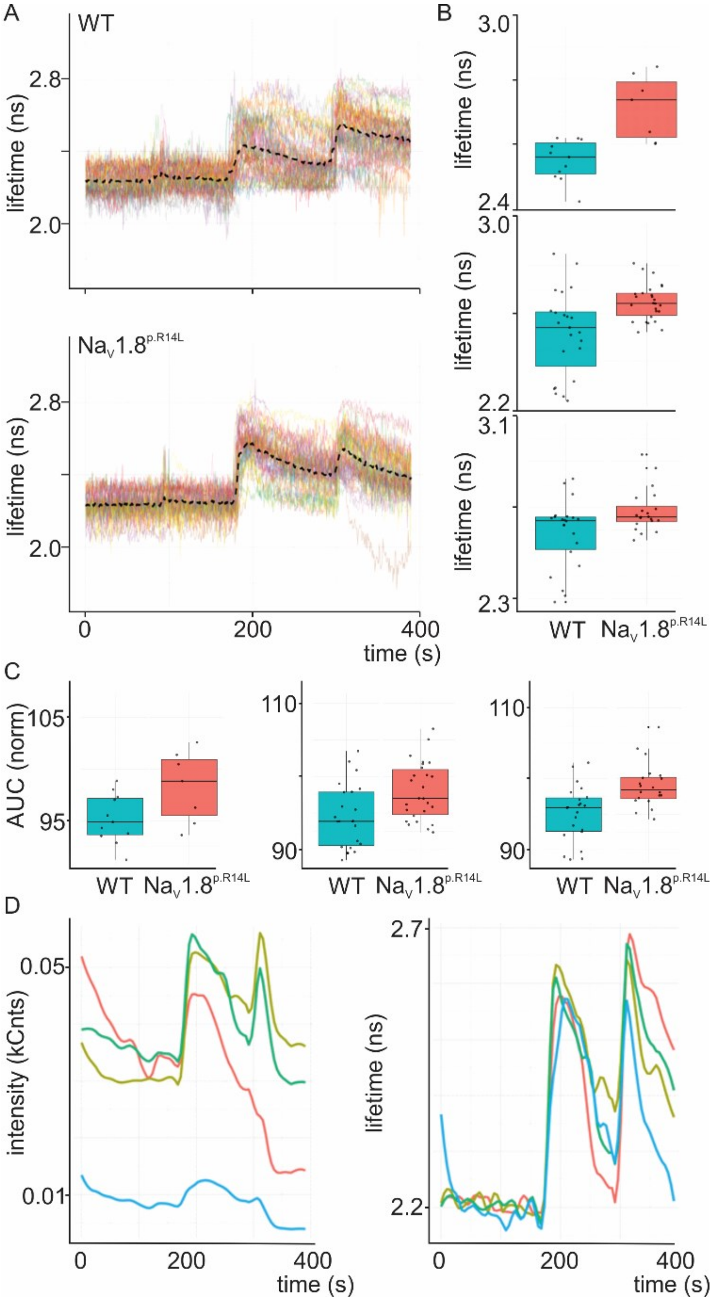

Figure S4: Additional Ca^{2+} imaging data. (A) Time-resolved fluorescence lifetime traces from individual regions of interest (ROIs) in cultured thoracic T1-T12 sympathetic neurons from WT (top) and Nav1.8$^{p.R14L}$ mice (bottom). Each colored trace represents one ROI; the black dashed line shows the average trace. **(B)** Boxplot of peak fluorescence lifetime values following carbachol stimulation from three different pairs of mice. Blue represents wild-type mice and red represents Nav1.8$^{p.R14L}$ mice. Each dot is an individual ROI measurement. **(C)** Area under the curve (AUC) analysis of lifetime values during the defined cholinergic stimulation window (170–260 s), normalized to each ROI's own pre-stimulus baseline (150–168 s), from three different pairs of mice. Blue represents WT mice and red represents Nav1.8$^{p.R14L}$ mice. Each dot is an individual ROI measurement. **(D)** Representative time-resolved fluorescence lifetime traces (right) and intensity traces (left) from individual ROIs. While intensity readouts are susceptible to

photobleaching and manual stimulation artifacts, the fluorescence lifetime measures remain stable and reliable.

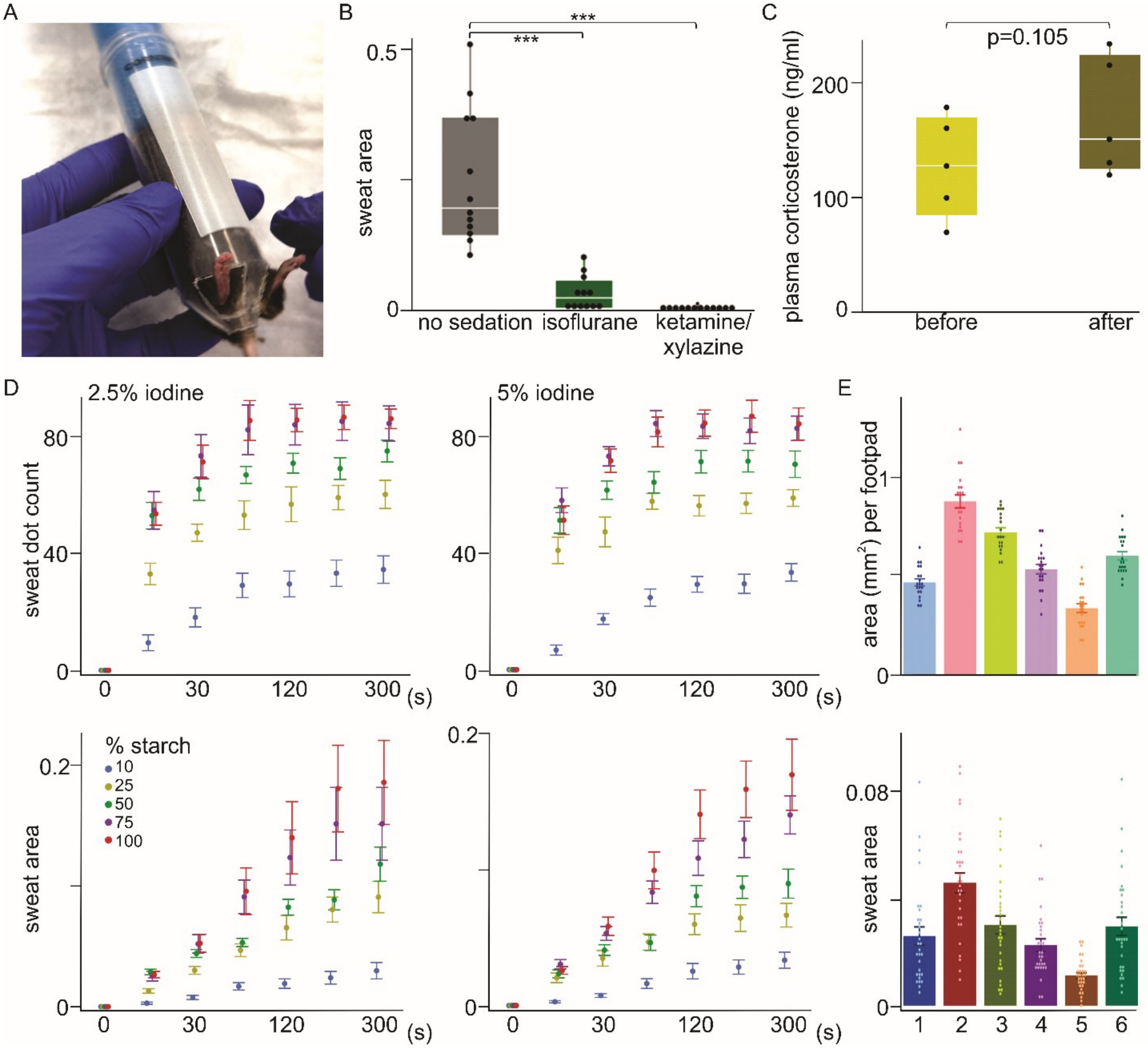

Figure S5: Optimization and validation of a non-sedated, restraint-based iodine-starch assay for dynamic sweat measurements. (A) Custom restraint device used for sweat quantification that preserves physiologically intact sympathetic tone. **(B)** Comparison of sweat output in awake versus anesthetized conditions demonstrates that both isoflurane and ketamine/xylazine significantly suppress sweating (***$p < 0.001$). **(C)** Plasma corticosterone levels measured before and after the assay show no significant elevation, confirming minimal stress induced by the restraint protocol. **(D)** Optimization of assay conditions using varying starch concentrations (10 %, 25 %, 50 %, 75 %, 100 %) under two iodine concentrations (2.5 % and 5 %) and two measures of sweat output, sweat droplet number and total sweat area. Although counting sweat droplets provided a straightforward measure, variability in droplet size both within and between mice could confound comparisons. Therefore, total sweat droplet area was quantified, providing a more accurate assessment of sweat output across samples. Optimal signal-to-noise was achieved with 2.5% iodine and 100 % starch. **(E)** Correlation between sweat area and footpad surface area across all six footpads per hind paw, confirming anatomical dependence of sweat output.

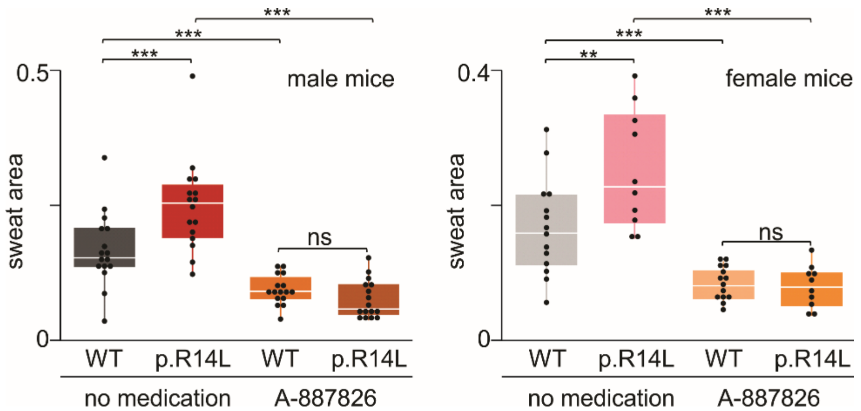

Figure S6: Evaluation of the contribution of Na$_V$1.8 to PIH pathophysiology with dynamic sweating measurements. Evaluation included a comparison of sweat area between WT (8 male, 7 female) and Nav1.8$^{p.R14L}$ (8 male, 5 female) mice, before and after treatment with 10 mg/kg A-887826 administered i.p. 30 min prior to the test. After treatment, the difference in sweat area between WT and Nav1.8$^{p.R14L}$ was no longer observed. Two-way ANOVA with specified Holm-Šídák-corrected pairwise comparisons and two-tailed Student's *t* tests and Welch's t-test were used. Significant difference with *$p < 0.05$, **$p < 0.01$ and ***$p < 0.001$.

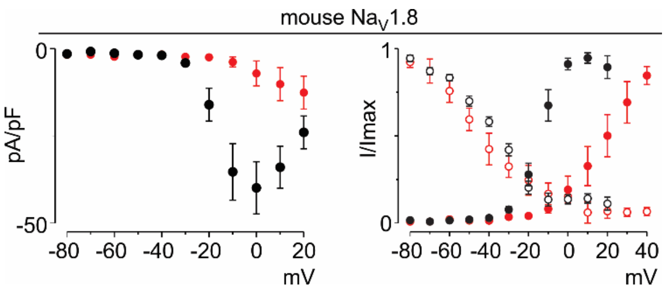

Figure S7: Electrophysiological characterization of the murine Na$_V$1.8$^{p.C1288W}$ variant. Whole-cell voltage-clamp recordings in ND7/23 cells expressing mouse Nav1.8$^{p.C1288W}$ reveal loss-of-function channel behavior. Compared to WT (black), the mutant channel (red) exhibits a marked reduction in peak current density (control/p.C1288W; n = 5 cells), consistent with decreased Na^+ conductance, as well as a large depolarizing shift in channel activation voltage. These findings corroborate the human electrophysiology data.

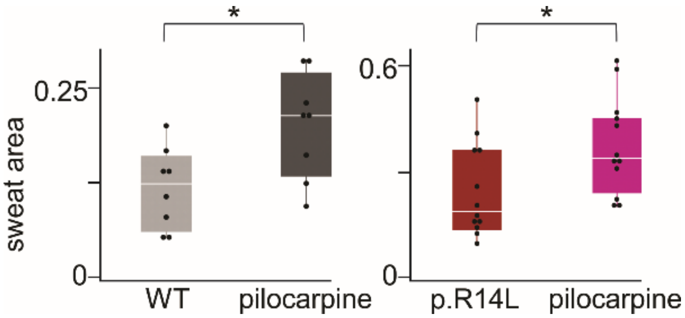

Figure S8: Pilocarpine induces sweating in both WT and Na$_V$1.8$^{p.R14L}$ mutant mice. Box plots show quantification of sweat response, measured as pixel intensity, following i.p. administration of pilocarpine (2.5 mg/kg) in WT (n = 4) and Nav1.8$^{p.R14L}$ (n = 6) mice. Pilocarpine significantly increased sweat output in both genotypes compared to untreated controls ($p < 0.05$). Data represent individual animals with each hind paw reported separately; horizontal bars indicate median values with interquartile range (*$p < 0.05$).

	WT				p.R14L				p-value
Resting membrane potential	-47.6	±	9.7	(39)	-43.0	±	9.0	(32)	0.042*
Input resistance	449.4	±	289.8	(39)	358.5	±	164.6	(32)	0.354
Membrane capacitance (pF)	23.1	±	8.7	(39)	25.1	±	10.4	(32)	0.569
Rheobase (pA)	11.5	±	8.0	(12)	17.0	±	8.3	(10)	0.045*
Current threshold (pA/pF)	0.5	±	0.3	(16)	0.6	±	0.4	(10)	0.262
AP threshold (mV)	-25.4	±	6.4	(16)	-16.8	±	8.1	(10)	0.006**
Time to AP threshold (ms)	93.9	±	12.8	(16)	92.3	±	11.6	(10)	0.763
Peak amplitude (mV)	80.7	±	14.9	(16)	69.5	±	12.5	(10)	0.060
Time of peak (ms)	95.2	±	12.8	(16)	93.8	±	11.6	(10)	0.786
AHP amplitude (mV)	-16.4	±	6.8	(16)	-15.5	±	6.6	(10)	0.737
Time of AHP peak (ms)	104.6	±	13.3	(16)	99.9	±	18.0	(10)	0.455
Max rise slope (mV/ms)	104.6	±	59.6	(16)	53.7	±	30.0	(10)	0.023*
Time of max rise slope (ms)	94.4	±	12.8	(16)	93.0	±	11.7	(10)	0.774
Max decay slope (mV/ms)	-31.2	±	12.3	(16)	-24.5	±	9.9	(10)	0.160
Time max decay slope (ms)	96.4	±	12.9	(16)	95.3	±	11.9	(10)	0.830
Rise time (ms)	8.4	±	5.8	(16)	13.0	±	5.6	(10)	0.023*
Rise slope (mV/ms)	6.8	±	6.4	(16)	2.6	±	1.3	(10)	0.035*
Decay time (ms)	2.5	±	0.6	(16)	2.8	±	0.9	(10)	0.697
Decay slope (mV/ms)	-24.9	±	9.0	(16)	-19.9	±	7.9	(10)	0.169
Peak amplitude overshoot (mV)	35.7	±	11.1	(16)	28.0	±	7.2	(10)	0.063
AP half-width (ms)	3.0	±	0.8	(16)	3.5	±	1.0	(10)	0.123

Table S1: Current-clamp parameters of primary thoracic postganglionic sympathetic neurons isolated from WT and $Na_V1.8^{p.R14L}$ mice. Data are presented as mean ± SD, with the number of recorded cells indicated in parentheses. Cells were obtained from 12 WT mice and 7 $Na_V1.8^{p.R14L}$ mice (biological replicates). Statistical comparisons were performed using unpaired two-tailed Student's *t*-tests or Mann–Whitney tests as appropriate based on data distribution. $p < 0.05$ (*), $p < 0.01$ (**). Rheobase represents the minimal injected current required to elicit an action potential. Current threshold (pA/pF) is normalized to membrane capacitance. AP, action potential; AHP, afterhyperpolarization; SD, standard deviation.

Condition	Permeability (cm/s) ± SD		
	24 h	48 h	72 h
Water	$13.36 \pm 1.97 \times 10^{-4}$ (n = 12)	$12.97 \pm 2.10 \times 10^{-4}$ (n = 13)	$13.52 \pm 1.70 \times 10^{-4}$ (n = 14)
hAQP5	$30.31 \pm 11.23 \times 10^{-4}$ (n = 14)	$35.31 \pm 10.21 \times 10^{-4}$ (n = 17)	$34.15 \pm 8.85 \times 10^{-4}$ (n = 18)
$hAQP5^{p.A193V}$	$29.33 \pm 6.12 \times 10^{-4}$ (n = 14)	$42.92 \pm 7.77 \times 10^{-4}$ (n = 14)	$49.18 \pm 10.28 \times 10^{-4}$ (n = 15)

Table S2: Osmotic water permeability (Pf) of oocytes expressing WT hAQP5 or $hAQP5^{p.A193V}$ compared to nuclease-free water-injected controls at 24-, 48-, and 72-hours post-RNA injection, measured 60s after hypo-osmotic shock. Values are given as mean ± SD and number of oocytes used is indicated in brackets.

Supplementary video 1A – WT

Examples of timelapse overlay of brightfield and fluorescence intensity of wild-type **(1A)** and Nav1.8$^{p.R14L}$ **(1B)** neurons showing calcium imaging performed on cultures of mouse thoracic sympathetic neurons. Imaging was performed with a 40× magnification (HC PL APO CS2 40x/1.25 GLYC, NA 1.25) objective with a spatial resolution of 0.57 μm/pixel. Frames were acquired every 0.865 seconds for a total duration of 6 minutes and 31 seconds (453 frames). The exported video was rendered at 15 frames per second. Brightfield channels show neuronal morphology, while fluorescence overlays indicate intracellular calcium dynamics. Scale bar: 50 μm.

Supplementary video 1B – Mut

Examples of timelapse overlay of brightfield and fluorescence intensity of wild-type **(1A)** and Nav1.8$^{p.R14L}$ **(1B)** neurons showing calcium imaging performed on cultures of mouse thoracic sympathetic neurons. Imaging was performed with a 40× magnification (HC PL APO CS2 40x/1.25 GLYC, NA 1.25) objective with a spatial resolution of 0.57 μm/pixel. Frames were acquired every 0.865 seconds for a total duration of 6 minutes and 31 seconds (453 frames). The exported video was rendered at 15 frames per second. Brightfield channels show neuronal morphology, while fluorescence overlays indicate intracellular calcium dynamics. Scale bar: 50 μm.

Supplementary video 2A – FLIM timelapse WT

Examples of fluorescence lifetime pseudocolor timelapses of wild-type **(2A)** and Nav1.8$^{p.R14L}$ **(2B)** neurons showing calcium imaging performed on cultures of mouse thoracic sympathetic neurons. Imaging was performed with a 40× magnification objective (HC PL APO CS2 40x/1.25 GLYC, NA 1.25) with a spatial resolution of 0.57 μm/pixel. Frames were acquired every 0.865 seconds for a total duration of 6 minutes and 31 seconds (453 frames). Fluorescence lifetime changes are represented using a "Turbo" color lookup table, where warmer colors indicate higher intracellular calcium levels and a color bar (0-4 ns) is included. Scale bar: 10 μm.

Supplementary video 2B – FLIM timelapse Mut

Examples of fluorescence lifetime pseudocolor timelapses of wild-type **(2A)** and Nav1.8$^{p.R14L}$ **(2B)** neurons showing calcium imaging performed on cultures of mouse thoracic sympathetic neurons. Imaging was performed with a 40× magnification objective (HC PL APO CS2 40x/1.25 GLYC, NA 1.25) with a spatial resolution of 0.57 μm/pixel. Frames were acquired every 0.865 seconds for a total duration of 6 minutes and 31 seconds (453 frames). Fluorescence lifetime changes are represented using a Turbo color lookup table, where warmer colors indicate higher intracellular calcium levels and a color bar (0-4 ns) is included. Scale bar: 10 μm.
